# Quantitative proteomic comparison of stationary/G_0_ phase cells and tetrads in budding yeast

**DOI:** 10.1038/srep32031

**Published:** 2016-08-25

**Authors:** Ravinder Kumar, Sanjeeva Srivastava

**Affiliations:** 1 Department of Biosciences and Bioengineering, Indian Institute of Technology Bombay, Powai, Mumbai-400076, Maharashtra, India

## Abstract

Most of the microbial cells on earth under natural conditions exist in a dormant condition, commonly known as quiescent state. Quiescent cells exhibit low rates of transcription and translation suggesting that cellular abundance of proteins may be similar in quiescent cells. Therefore, this study aim to compare the proteome of budding yeast cells from two quiescent states *viz.* stationary phase/G_0_ and tetrads. Using iTRAQ (isobaric tag for relative and absolute quantitation) based quantitative proteomics we identified 289 proteins, among which around 40 proteins exhibited ±1.5 fold change consistently from the four biological replicates. Proteomics data was validated by western blot and denstiometric analysis of Hsp12 and Spg4. Level of budding yeast 14-3-3 proteins was found to be similar in both the quiescent states, whereas Hsp12 and Spg4 expressed only during stress. FACS (fluorescence-activated cell sorting) analysis showed that budding yeast cells were arrested at G_1_ stages both in tetrads as well as in stationary phase. We also observed that quiescent states did not express Ime1 (inducer of meiosis). Taken together, our present study demonstrates that the cells in quiescent state may have similar proteome, and accumulation of proteins like Hsp12, Hsp26, and Spg4 may play an important role in retaining viability of the cells during dormancy.

In natural ecosystems, starvation is one of the most common stress encountered by almost all microbial species. It is estimated that most, if not all, of the micro-organisms biomass in the world exists under nutrient-depleted condition^1^. Bacterial cells respond to hostile environments like nutrient deficiency and presence of toxic chemicals by forming inert structures commonly referred as bacterial spores, well known for their ability to resist physical and chemical challenge^2^,^3^,^4^,^5^.

Like prokaryotes, the eukaryotic species also form differentiated cells or spores capable of survival during extended periods of nutrient(s) deficiency. In eukaryotes, fungal species are well known for their ability to form spores capable of surviving under environmental conditions, which does not support rapid growth like, limiting supply of essential nutrients such as carbon and nitrogen. Eukaryotes such as *Saccharomyces cerevisiae*, respond and cope-up starvation by ceasing growth and entering into a non-proliferating state referred to as stationary phase^6^,^7^,^8^ or by forming spores^9^,^10^,^11^. Cells in stationary phase differentiates in ways that allows maintenance of viability for extended periods without added nutrients but retain the ability to resume growth promptly when appropriate nutrients become available. Quiescent state is highly regulated and programmed phenomenon^11^,^12^,^13^.

During entry into the quiescent state, microorganisms undergoes morphological and physiological changes that allows them to resist the effects of environmental stresses. Both prokaryotic and eukaryotic cells, such as bacteria, yeast, and neuronal cells, can persist in the quiescent state for years^11^,^14^. Availability of genomic tools such as microarray allowed detailed characterization of global transcripts changes in yeast cells during the stationary-phase^15^.

Apart from the implications for basic cell physiology, quiescent state has prominent role in biomedical, environmental research and agriculture, which affects the whole human race (as more than 70% of plant pathogen are fungal in nature, which can persists in the field, in form of spores or as stationary phase cells). Therefore, information about the processes of survival, entry and exit from stationary-phase, may lead to the development of better treatment strategies. Quiescent yeast cells may also provide an excellent model system for aging since cells in stationary-phase cultures have been found to possess a shorter replicative life-span, mimicking aging in non-dividing cells of other organisms^16^. Further, most of the microorganisms exist in an un-culturable quiescent state, which makes it difficult to understand the contribution of these species to critical environmental processes such as carbon fixation. Thus, the ability to revoke the cell cycle process in these organisms is an important beginning towards understanding their role in broader context of environment.

Budding yeast cells, at stationary phase, shared key features of spores, including increased thermo-stability, low metabolic activity, reduced transcription and translational activity, resistance to various environmental stresses etc. When stationary phase and spores are kept in nutrient rich media both resume growth and lose their characteristics, which are hallmarks of spores or stationary cells^11^. This suggests that the proteome of cells of these two dormant stages may be similar. Thus aim of our study was to compare the proteome of tetrads and stationary phase cells using gel-free iTRAQ-based quantitative proteomics^17^. We identified around 289 proteins common in four independent biological replicates. Out of these 289 proteins, around 40 proteins showed fold change equal or more than ±1.5 and less than dozen showed fold change of ±4, suggesting that although abundance of few proteins changes during these two quiescent stages but overall cellular abundance does not change significantly. Cellular abundance of budding yeast Bmh1 (Brain Modulosignalin Homologue) and Bmh2 were found to be similar in cells at G_0_/stationary phase and tetrads, which are important for cell viability under stress and during quiescent stage. Many of the proteins involved in pathways that are important for entry, maintenance and exit of quiescent stages, possess Bmh interacting motifs. We validated our proteomics data by western blot and denstiometric analysis of Hsp12 and Spg4 along with budding yeast 14-3-3 proteins. Under both quiescent conditions, cells remain arrested in G_1_ and stationary phase/G_0_ cells lacks Ime1. Hsp12 is important for survival of cells under diverse stress conditions. Our data suggests that cellular abundance of proteins may be similar in quiescent cells and many of these proteins are important for survival under environmental stress conditions.

## Results

### Does a budding yeast cell possess similar proteome during tetrads and stationary phase/G_0_?

**Figure 1:**
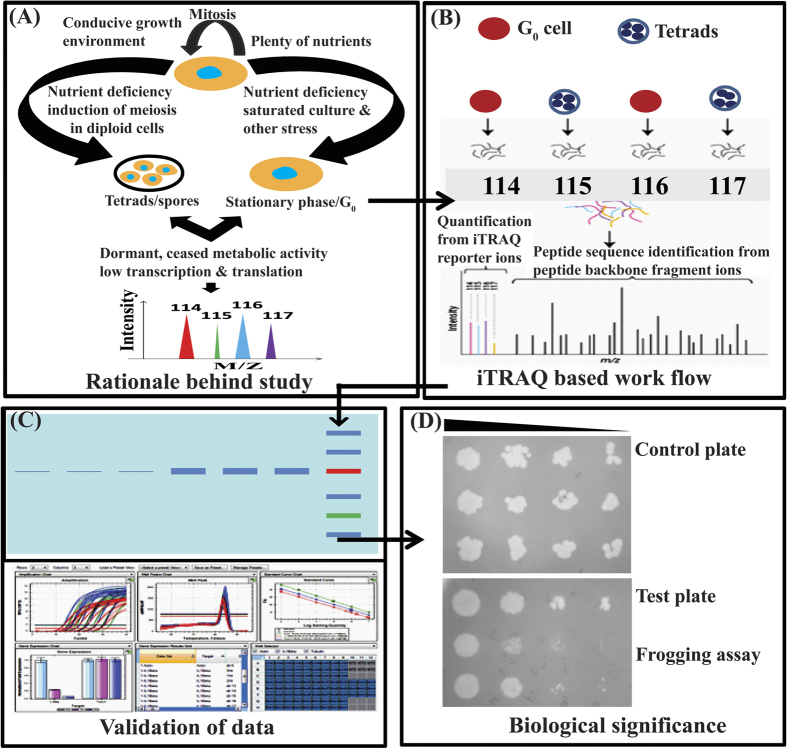
Schematic representation of rationale and the experimental strategy used for comparative analysis of quiescent cells. (**A**) Rationale behind the study, (**B**) work flow of iTRAQ based proteomics, (**C**) validation of proteomics data by western blot and RT-PCR, and (**D**) biological significance of selected proteins.

Quiescent or dormant state allows microbial cells to survive and retains viability for significant period of time during unfavourable conditions, including deficiency of essential nutrient(s). Budding yeast cells respond to nutrient deficiency by entering into either of two distinct resting states *viz* stationary phase or G_0_^18^ and the spore or tetrads^10^,^11^. Since tetrads or spores share numerous unique attributes of stationary phase cells, hence tetrads or spores also represent quiescent cells similar to G_0_/stationary phase cells^19^. Owing to shared behaviour of low rate of transcription, translation and key properties of quiescent cells, it is anticipated that the budding yeast cells at resting phase during tetrads and stationary phase/G_0_ may have similar proteome i.e. cellular abundance of proteins may be similar or vary with slight variations. Present work was designed to test this hypothesis and deduce reliable and relative inference. A schematic showing rationale and work-flow for objectives proposed in this study is shown in Fig. 1.

### Stationary phase/G_0_ arrest of cells

**Figure 2:**
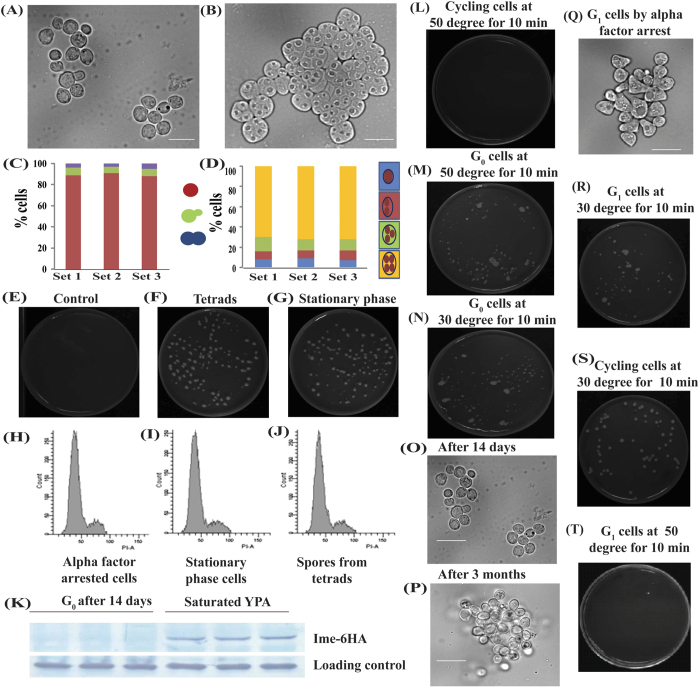
Validation of G_0_/stationary phase cells and tetrads. Morphology of (**A**) G_0_/stationary phase cells and (**B**) sporulating cells with tetrads, triads, dyads. Efficiency of (**C**) G_0_/stationary phase arrested cells (along with cartoon presentation, showing relative abundance of cells with given morphology i.e. unbudded, small budded and large budded), and (**D**) sporulation efficiency (along with carton presentation showing relative abundance of tetrads, triads, dyads and non sporulating cells). (**F**) Tetrads and (**G**) stationary phase/G_0_ cells retain viability even after incubation for three month in spent out media and water, respectively, while cycling cells loose viability (**E**). (**H**) Ploidy of control cells arrested at G_1_ phase by alpha factor arrest. Ploidy of (**I**) stationary phase cells, and (**J**) spores collected on zymolase treatment of tetrads, triads, dyads. (**K**) Stationary phase/G_0_ cells does not express Ime1 (left panel) while cells in saturated culture in YPA express Ime1 (right panel). (**L**) Cycling cells incubated at 50 °C for 10 min. (**M**) G_0_ phase cells incubated at 50 °C for 10 min. (**N**) G_0_ phase cells incubated at 30 °C for 10 min. Morphology of stationary phase cells (**O**) on 14^th^ day after incubation in YPD and (**P**) after three months in spent out media. (**Q**) Morphology of alpha factor arrested cells at G_1_, (**R**) G_1_ cells incubated at 30 °C for 10 min, (**S**) cycling cells at 30 °C for 10 min, and (**T**) G_1_ cells incubated at 50 °C for 10 min. Scale bar represents 5 μM.

Cells entering stationary phase followed a characteristic growth curve^11^,^20^,^21^. During entry of stationary phase, the cells became relatively round and unbudded. We also observed a characteristic growth curve using diploid SK1 background strain (data not shown). We further checked the morphology of cells after fourteen days (Fig. 2A). For protein extraction, cells were arrested at stationary phase in four biological replicates (Fig. 2C) (data is shown for only three batches of culture). Morphology of stationary phase cells are shown in Fig. 2A in which most of the cells were round and unbudded while some cells were with big buds. Fractions of cells, with different morphologies at stationary phase are shown in Fig. 2C. Our data for stationary phase is in accordance with previous study^22^, which showed that cells can exist at stationary phase, irrespective of different phases of cell cycle.

SK1 background is routinely used in labs for studying meiosis and sporulation owing to ease and efficiency of sporulation in this background^23^. Tetrads that we used for protein extraction are shown in Fig. 2B. Sporulation efficiency was checked by counting sporulating cells under microscope, and culture with more than 90% sporulation was used for protein extraction. Relative percentage of tetrads, triads, dyads and non-sporulating cells are shown in Fig. 2D. For protein extraction, sporulation was performed in four batches (Fig. 2D).

### Thermo-stability of cells arrested at stationary phase/G_0_

Apart from round unbudded morphology, cells arrested at stationary phase/G_0_ also showed thermo-tolerance/resistance compared to cycling cells^13^,^24^. Thermal tolerance test for stationary phase cells was performed using procedure explained in materials and methods. Our data shows that cells which were used for protein extraction also acquired thermal stability or tolerance and remained viable even after thermal shock for 10 min at 50 °C (Fig. 2M) as compared to the cycling cells (Fig. 2L). Percentage of cells which retains viability after thermal heat shock was around 90%, which is in accordance with previous study^24^.

Since stationary phase cells as well as cells at G_1_ phase appeared round and unbudded. Thus thermal stability assay was also performed by taking alpha factor arrested G_1_ cells to confirm that only unbudded stationary phase cells survive thermal shock while round cells arrested at G_1_ phase does not survive thermal shock. Alpha factor arrest was performed as described in materials and methods^25^. Efficiency of alpha factor arrest was checked under bright microscope and alpha factor arrested cells at G_1_ (shmooed structure) shown in Fig. 2Q. These cells along with normal cycling cells were incubated in water bath maintained at 50 °C for 10 min and known number of cells were plated on YPD plates and incubated at 30 °C for 72 hour. Our data showed that cells at G_1_ phase lack thermo stability unlike that of G_0_ cells, which retains viability even after thermal shock at 50 °C for 10 min (Fig. 2M,T). Our results are in accordance with previous study^24^. We did not obtain even single colony for alpha factor arrested cells at G_1_ after thermal shock at 50 °C for 10 min (Fig. 2T), while untreated G_1_ phase cells retained viability (Fig. 2R) along with cycling untreated cells used as control (Fig. 2S). This suggests that G_1_ phase cells lack thermal tolerance in contrast to stationary phase or G_0_ cells.

### Viability of stationary phase cells kept in nutrient deficient condition

Apart from thermal tolerance, stationary phase cells are able to retain viability for extended period of time even when kept in nutrient deficient media like spent out media or even in sterile water^26^. Cells were incubated in spent out media for three months to analyse their viability to ensure that the cells remain in stationary phase. Our results showed that the cells in a culture used for protein extraction retains viability even when kept in spent out media for around three months at room temperature (Fig. 2G). This observation along with other tests confirmed that the cells used for protein extraction for proteomics investigations were indeed at resting phase. We also demonstrated that like stationary phase, tetrads also retains viability in sterile water even after three months (Fig. 2F). We further checked the morphology of stationary phase cells after incubation in spent out media for three months (Fig. 2O,P). Therefore, our data showed that both stationary phase cells as well as tetrads retain viability even in complete absence of nutrients. These observations also suggested the shared properties of dormant or quiescent cells.

### FACS analysis of stationary phase cells

So far it is known that there is decreased rate of transcription and translation in resting cells whether it is spores in tetrads or stationary phase cell. Both resting phase cells also shared many attributes unique to them and absent in normal cycling cells^11^. Further, it is known that most of the cells in stationary phase are round and arrested at G_0_ phase. To further test and compare ploidy of stationary phase cells, FACS analysis was performed for both stationary phase and spores collected on zymolase treatment of tetrads, triads, dyads. Our data showed that in both the cases i.e. stationary phase and spores, cells were arrested at G_1_ phase before START point (Fig. 2I,J), which is in accordance with previous studies for stationary phase cells^6^,^27^. Alpha factor arrested cells at G_1_ were used as positive control (Fig. 2H). For this particular experiment, stationary phase arrest was performed in haploid wild type strain, which allowed comparison of FACS data between haploid spores and alpha factor arrested Mat a strain.

### Stationary phase cells do not express Ime1

During stationary phase/G_0_ arrest of cells, we could not detect any tetrads or sporulating cells in culture of wild type strain grown in YPD for up to two weeks, although we were able to detect significant proportion of sporulating cells in saturated culture in YPA after one week. We found approximately 15% sporulating cells in one week old saturated YPA culture (data not shown). It is known that induction of meiosis and sporulation is controlled by Ime1^28^. Therefore, we tested the presence of Ime1 in 14 days old culture from YPA and YPD. Western blot demonstrated that diploid cells in saturated culture in YPA expressed Ime1 while cells in YPD lacked Ime1 (Fig. 2K), supporting previous studies that reported the absence of glucose and presence of non-fermentable carbon (example acetate) source is important for expression of Ime1 and induction of meiosis^29^,^30^,^31^. Non-fermentable carbon source such as acetate is important for expression of Ime1^32^,^33^, which is important for expression of early, middle and late meiotic genes through Ime2.

### Cellular abundance of proteins is similar in quiescent cells

**Figure 3:**
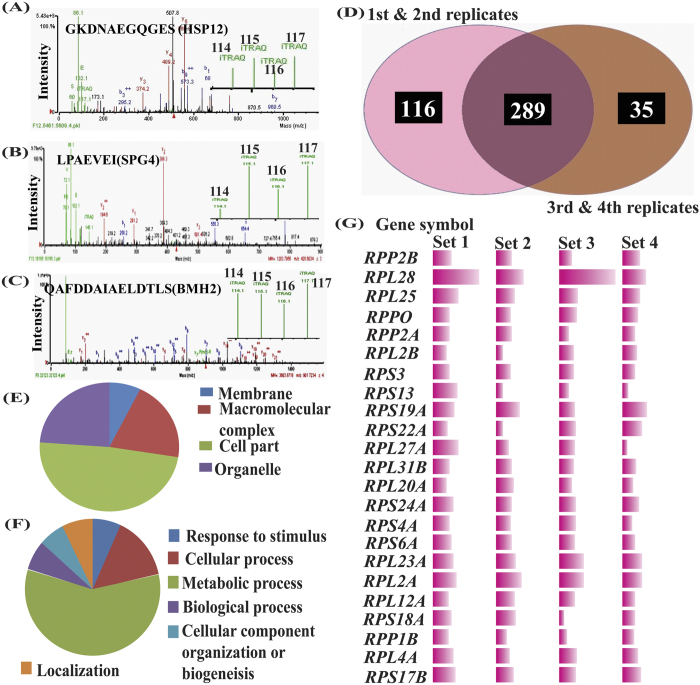
Bioinformatic analysis of proteins identified in iTRAQ based comparison of G_0_/stationary phase and tetrads. Spectra of one of the peptide of (**A**) HSP12, (**B**) SPG4 and (**C**) BMH2 along with intensity of reporter ions showing relative abundance of proteins. (**D**) Venn diagram showing number of protein common in biological replicates. Grouping together of proteins, based on (**E**) cell components and (**F**) biological functions. (**G**) Ribosomal proteins with gene symbol and fold change across four biological replicates (complete list of proteins detected in iTRAQ based analysis are given in Table S5).

For this proteomic study, culture was grown independently on different days and protein extraction was performed in four biological replicates (in Fig. 2C,D) as described previously^34^. Despite the fact that stationary phase/G_0_ cells and tetrads possess thick cell wall, which makes protein extraction difficult, we were able to identify 289 proteins in iTRAQ experiment, which were common in four biological replicates. We maintained high stringency during iTRAQ data analysis and only proteins with 1% FDR (false discovery rate) and 60% SPI were considered for further analysis. Since this was a quantitative proteomic investigation, only those proteins showing differential expression using iTRAQ labels were identified. Out of these 289 proteins approximately around 40 proteins showed cellular abundance equal or more than 1.5 fold (Table 1). Spectra of one of the peptide of HSP12, SPG4 and BMH2 along with intensity of reporter ions (iTRAQ labels) are shown in Fig. 3A–C, respectively. Parameters used for analysis of iTRAQ data are presented in Table S3. List of proteins identified in four biological replicates and proteins common across all sets are shown in Table S4 (through Venn diagram, Fig. 3D). Fold change and trend of some of the ribosomal proteins are shown in Fig. 3G, while complete list (in the form of heat map) of ribosomal proteins detected across all biological replicates are shown in Table S5.

To further test the abundance of proteins in tetrads and stationary phase, protein summary details were exported and absolute intensity obtained from each peptide was averaged using R programme. These intensity values were further averaged to calculate the average intensity of all proteins across various biological replicates. The average intensity was plotted against number of proteins. For visual depiction of protein abundance, graph was plotted between abundance and number of proteins in stationary phase and tetrads (Fig. S2A,B). Overlap of protein abundance between tetrads and stationary phase is shown in Fig. S2C. We further made scatter plot showing protein abundance in stationary phase/G_0_ and tetrads along X and Y axis, respectively (Fig. S2D). T-test was performed using average intensities (as mentioned above). Values of t-test for 289 proteins common in four biological replicates are shown in Table S6. Proteins for which *p*-value was less than 0.05 or which were statistically significant and having fold change more than 1 fold across all biological replicates are highlighted in Supplementary data (Table S6). Out of 289 proteins common in four biological replicates, around 89 proteins were statistically significant out of which less than 13 showed fold change more than 1.5 (across all biological replicates).

Bioinformatic analysis of proteins identified in iTRAQ-based comparison of G_0_/stationary phase and tetrads was carried out (using PANTHER, freely available online software http:// www.pantherdb.org/pathway/) to group the proteins based on biological processes, molecular functions, cellular components and distribution of proteins into different classes to understand their biological relevance. Results of bioinformatic analysis showed that proteins from diverse cellular processes and pathways were identified in our study. PANTHER grouped all the proteins into ten groups based on their molecular functions (Fig. S1A). When grouped together based on class of proteins, all proteins were broadly classified into 21 groups (Fig. S1B) but only four groups were observed when proteins were distributed based on cell component viz., membrane (7.3%), macromolecular complex (20.2%), cell part (48.6%) and organelle (23.9%) (Fig. 3E). When same proteins were analysed based on biological processes, they formed six major groups, including biological regulation (6.8%), cellular component organization or biogenesis (6.2%), cellular process (14.7%), localization (7.4%), metabolic process (78.2%) and response to stimulus (6.8%) (Fig. 3F).

### Western blot and denstiometric analysis of Hsp12 and Spg4

**Figure 4:**
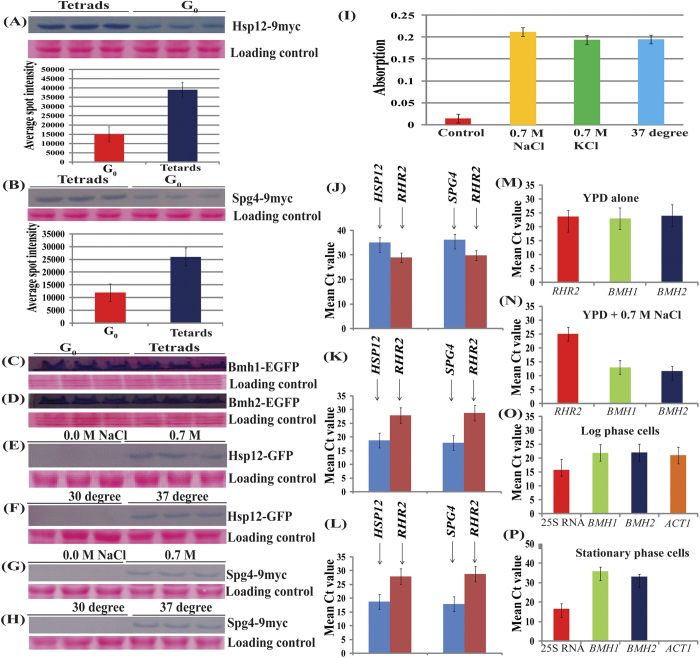
Increased abundance and presence of Hsp12 and Spg4 in tetrads and in cells under stress. Western blots and denstiometric plots showing the quantitative expression of (**A**) Hsp12-9myc, (**B**) Spg4-9myc in tetrads and G_0_/stationary phase. Lanes, 1–3: tetrads and 4–6: G_0_/stationary phase. 9myc was detected using anti-myc antibodies. Volume intensity was calculated for each band using iQTL software and average volume intensity was used to plot the bar graph with standard deviation (n = 3). (**C**) Level of Bmh1-EGFP, and (**D**) Bmh2-EGFP in G_0_/stationary phase (left) and tetrads (right). Proper loading of protein are shown by Ponceau stained blots. Presence of Hsp12 (**E,F**) and Spg4 (**G,H**) in cells under stress (right) and during normal growth regime (left). (**I**) Intensity of signal coming from normal cells (red bar), in presence of 0.7 M NaCl (yellow bar), 0.7 M KCl (green bar) and cells grown at 37 °C (blue bar). The numbers of cells were kept equal in all the cases and absorption was performed at 488 nm. Increased expression of *HSP12* and *SPG4* in cells under stress due to (**K**) 0.7 M NaCl and (**L**) 0.7 M KCl compared to (**J**) control cells, where cells were harvested at OD_600nm_ 1.0 in all the cases. Expression of *BMH1* and *BMH2* in (**M**) control cells in YPD, (**N**) in YPD with 0.7 M NaCl. *RHR2* also known as GPI was used as internal control. Expression of *BMH1* and *BMH2* in log phase cells in (**O**) YPD and (**P**) stationary phase/G_0_ cells on 14^th^ days after incubation in YPD. 25 S RNA was used as internal control.

Validation of proteomics findings was performed by western blot and denstiometric analysis of Hsp12 and Spg4. Our data showed increased abundance of Hsp12 (Fig. 4A) and Spg4 (Fig. 4B) in tetrads. Fold changes obtained for both Hsp12 and Spg4 were comparable, and in accordance with iTRAQ data for these proteins. We further compared the level of Bmh2 along with Bmh1 in G_0_ and tetrads. Our western blot analysis showed that the level of Bmh1 (Fig. 4C) and Bmh2 (Fig. 4D) proteins were similar in G_0_/stationary phase and tetrads. Ponceau stained blot were used as loading control. Specificity of antibodies was checked separately. Recent study also showed an accumulation of Bmh1 in spores in tetrads^35^. Thus it will be interesting to test whether Bmh2 also localizes in spores in tetrads. Such localization studies will certainly help in defining biological significance of these proteins in quiescent state. Our western blot data for Bmh2 was in accordance with iTRAQ data.

Effect of thermal and osmotic stress on cellular abundance of Hsp12 and Spg4 was checked using western blot. Our data showed that cellular abundance of Hsp12 (Fig. 4E,F) and Spg4 (Fig. 4G,H) increased during stress while normal cells lack these proteins. The western blot data was further supported by observations from plate reader where readings showed increased abundance of Hsp12-GFP in cells under stress as compared to the cells under normal condition (Fig. 4I). Ponceau stained blot was used as loading control. Our present observations are supported by previous studies from literature^36^.

### Induction of *HSP12* and *SPG4*

The presence of Hsp12 and Spg4 in stress conditions and their absence in normal cell culture was confirmed by western blots. Further, previous studies also showed induction of genes coding for Hsp12 and Spg4 under osmotic stress^37^ suggesting that *HSP12* and *SPG4* are expressed only during stress. We observed increased abundance of *HSP12* and *SPG4* mRNA in cells under osmotic stress induced by addition of 0.7 M NaCl (Fig. 4K) and 0.7 M KCl (Fig. 4L). Our RT-PCR data is in accordance with previous reports for *HSP12* 38 and *SPG4*^37^,^38^. Our observation is supported by previous study, which showed absence of *HSP12* mRNA in normal cells using northern blot^36^, *RHR2* (also known as *GPP1*) was taken as internal control^39^. Apart from stress during stationary phase, elevated temperature also leads to induction of *HSP12*^36^, *HSP26* and; *HSP26* follows expression pattern similar to that of *HSP12*. We also detected Hsp26 (protein known to be present in stationary phase cells) in our proteomics investigation of stationary phase and tetrads. The level of *RHR2* remains similar in osmotic stress induced by NaCl and KCl.

### Transcription of *BMH1* and *BMH2*

Several genome-wide expression studies using DNA microarrays have showed that expression of budding yeast 14-3-3 proteins are affected by various types of stress^15^. Thus, we were interested to examine the expression of *BMH* genes under osmotic stress and quiescent state. Our RT-PCR data demonstrated that level of both *BMH1* and *BMH2* increased in cells under osmotic stress induced by addition of 0.7 M NaCl (Fig. 4N) compared to control cells (Fig. 4M). Our data for RT-PCR for *BMH1* and *BMH2* is in accordance with previous reports^15^,^40^. Level of *RHR2* remains almost similar in both salt treated and untreated culture^36^. Results obtained from RT-PCR showed that unlike osmotic stress, there is decreased transcription of budding yeast 14-3-3 proteins in cells at stationary phase (Fig. 4P) compared to log phase cells (Fig. 4O). Our data is in accordance with previous report, which demonstrated around three fold reduction in mRNA level for *BMH1*. Thus expression of *BMH1* and *BMH2* is repressed in stationary phase cells. Further, it might be possible that low mRNA for budding yeast 14-3-3 may be due to decrease in overall transcription^41^,^42^ and slow degradation of total RNA in quiescent cells is due to increased abundance of RNAse^13^,^42^.

It is also interesting that we did not find any signal for *ACT1* in case of stationary phase cells, suggesting complete repression of *ACT1* in stationary phase cells (Fig. 4P). Our RT-PCR data is in accordance with previous northern blot study, which also showed absence of *ACT1* mRNA from cells of stationary phase using northern blot^36^. 25S RNA was used as an internal control as the cellular abundance of this RNA species remains almost unchanged in cells at log phase and in stationary phase cells^43^.

### Budding yeast 14-3-3 are important for cell survival under osmotic stress and quiescent state

**Figure 5:**
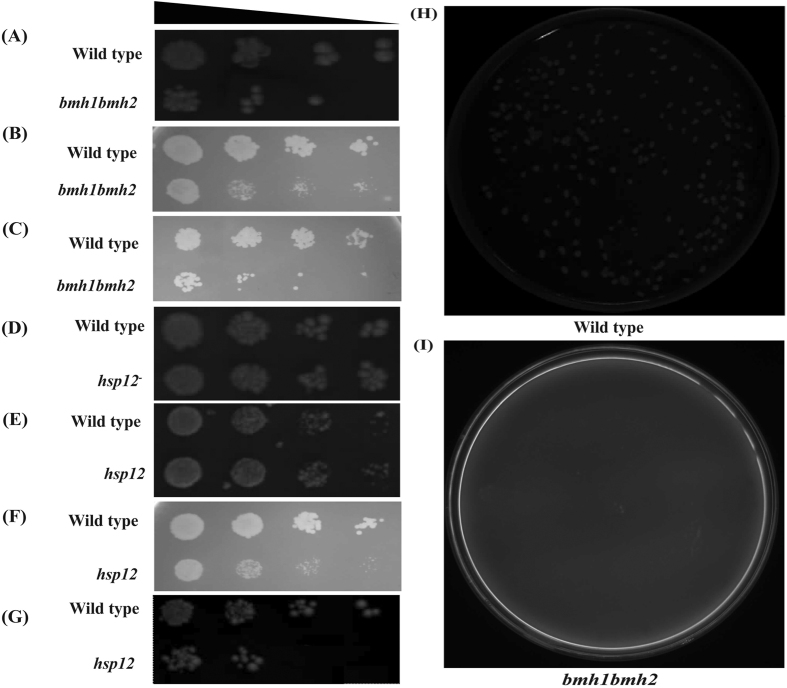
Role of yeast 14-3-3 and Hsp12 in cell survival. Growth of wild type and *bmh1bmh2* strain on (**A**) YPD plate, (**B**) YPD plate with 0.7 M NaCl, and (**C**) YPD plate with 0.7 M KCl. Growth of *hsp12* under (**D**) normal condition, (**E**) at 37 °C, (**F**) YPD along with 0.7 M NaCl and (**G**) YPD along with 0.7 M KCl. (**H**) Wild type cells retained viability during quiescent stage while (**I**) *bmh1bmh2* strains lost viability during quiescent states.

Similar cellular abundance of budding yeast 14-3-3 proteins in quiescent cells showed that these proteins may play important role in cell survival under stress. Thus we further investigated the significance of Bmh1 and Bmh2 in cell survival under osmotic stress induced by 0.7 M NaCl and 0.7 M KCl. Our frogging assay showed that *bmh1bmh2* strains become highly sensitive for osmotic stress compared to wild type control (Fig. 5A–C). Our data showed that even wild type cells were also sensitive towards osmotic stress (Fig. 5B,C) but to lesser extent compared to *bmh1bmh2* strain. We further checked the role of Bmh proteins in viability of quiescent cells and our results suggested that Bmh proteins are important for retention of viability in quiescent cells (Fig. 5H,I). Therefore, our data demonstrated that budding yeast 14-3-3 proteins may be important for cell viability under stress.

### Role of Hsp12 in stress, sporulation, spore viability and meiotic progression

Induction and presence of Hsp12 during stress showed likely involvement of Hsp12 in cell viability during stress. Thus we checked the role of Hsp12 in cell growth and survival under different stress environment. Our data showed that Hsp12 is important for growth of cell under stress. Compared to wild type strain, the mutant strain showed significant and dramatic growth reduction on YPD plate with 0.7 M NaCl (Fig. 5F) or 0.7 M KCl (Fig. 5G) used for induction of osmotic stress. Osmotic stress induced by addition of either NaCl or KCl exhibited similar effect on growth of *hsp12*^*−/−*^ strain. This suggests that whatever the cause of osmotic stress, mutant strain with deleted *HSP12* have similar effect on growth. We further showed that thermal stress (growth at 37 °C) does not affect growth of wild type as well as *hsp12*^*−/−*^ mutant strain (Fig. 5E). Our observations are supported by previously published work^44^. Taken together, it can be concluded that Hsp12 is important for cell survival under diverse stress conditions, including osmotic stress.

**Figure 6:**
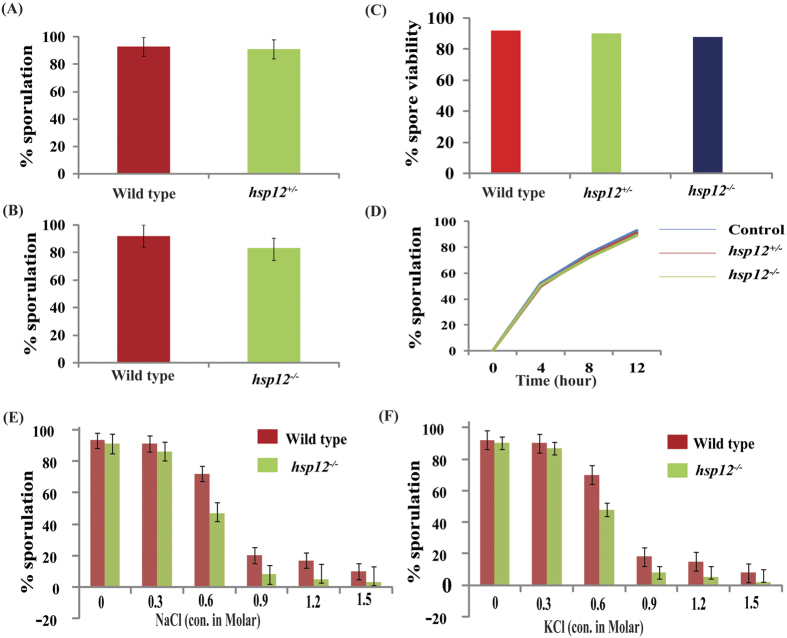
Role of Hsp12 in meiosis. Efficiency of sporulation in (**A**) *hsp12*^+/−^ and (**B**) *hsp12*^−/−^. (**C**) Percentage of spore viability and (**D**) meiotic progression in *hsp12*^−/−^ strain and wild type control. Note, for spore viability minimum 40 tetrads were dissected. The meiotic progression was performed thrice (representative data is shown for only one set). Sporulation efficiency in *hsp12*^−/−^ along with wild type control in presence of different concentration of (**E**) NaCl (**F**) KCl. Concentration of salt used is mentioned in figure.

To obtain deeper insight into the biological significance of increased abundance of Hsp12 in tetrads, meiotic induction was performed in *HSP12* mutant strain along with wild type control. Our results suggests that deletion of either single allele (Fig. 6A) or both the alleles (Fig. 6B) of *HSP12* does not affect sporulation. Even spore viability was found to be similar in both strains; homozygous as well heterozygous for *HSP12* compared to wild type control (Fig. 6C), suggesting that Hsp12 may not have any direct role in meiosis and sporulation (here we mean role in chromosome segregation, crossing over). However, the proteins may be involved in overcoming dietary stress (during period when cells are kept in sporulating media). We also tested the meiotic progression in wild type as well as mutant strains but no significant difference was observed in meiotic progression in wild type as well as in mutant strains (Fig. 6D).

Further, to investigate the possible role of Hsp12 in meiosis, we introduced different concentration of NaCl (Fig. 6E) and KCl (Fig. 6F) to study the effect of osmotic stress on sporulation in wild type as well as in *hsp12*^*−/−*^. Our data showed that mutant strain is more sensitive towards osmotic stress as compared to wild type strain. It is important to mention that osmotic stress induced by addition of either NaCl (Fig. 6E) or KCl (Fig. 6F) have similar effect on sporulation suggesting that cells undergoing meiosis may exhibit similar type of osmotic stress, which is irrespective of chemical identity of agent used for induction of osmotic stress. This further showed an important role of Hsp12 in overcoming stress during incubation of cells in sporulating media. Our data suggests that the reduction in sporulation efficiency is much more significant in mutant compared to wild type strain. Upto 0.3 M NaCl or KCl, there was no significant difference observed in sporulation efficiency in both wild type as well as in mutant strain but there was sudden and significant change in the rate at which sporulation efficiency falls in wild type and mutant strains. Additionally, we observed that decrease in sporulation or sporulation efficiency followed similar trend on addition of either KCl or NaCl.

## Discussion

In natural environment, supply of nutrients and other life supporting factors (like water, air, temperature, osmolarity) are highly discontinuous and exists for very brief period of time. In such a scenario microorganisms suffer nutrient(s) scarcity, which varies greatly with respect to time. As a result, most of the microbial cells under natural conditions, including prokaryotes or bacteria^45^ and unicellular eukaryotes (example budding yeast) spends most of their time in G_0_ or stationary phase^12^ or by forming spores^7^. Only when prevailing environment become conducive for growth, these cells exit dormant stage and enter phase of rapid growth. Similarly, budding yeast cells undergo rapid growth in environment with ample supply of fermentable carbon (example dextrose) and nitrogen. During limitation of either fermentable carbon or absence of essential elements like nitrogen; budding yeast shifts itself from mitotic to meiotic mode whose end products are four spores in ascus^9^ or enters stationary phase^6^,^7^,^8^. These dormant stages allows survival of yeast under stressful conditions^1^, including osmotic stress, high temperature^24^, presence of toxin in immediate surroundings.

The advancement in genomics made study of quiescent cells much more tractable and allowed the characterization of global changes in transcripts abundance that occurs when yeast cells entered the stationary phase^15^,^46^. Similarly, transcriptomics study in *C. albicans* showed that there is general decrease in overall mRNA, transcription of several genes increased in post-diauxic shift phase as well as during stationary phase. Genes whose product(s) involved in gluconeogenesis, stress resistance, adherence, DNA repair and ageing were expressed at higher levels at and beyond post-diauxic shift phase. Apart from that the microarray data showed that genes associated with virulence, drug resistance and cell-wall biosynthesis were expressed only at stationary phase^47^. Compared to transcriptomics, proteomics analysis of stationary phase cells is still lagging behind with only few published reports^20^,^48^ and thus providing an opportunity for contemporary biologist to look into the proteome of stationary cells, which will certainly flourish with wealth of mechanistic information, thus helping in better understanding of the physiology of dormant cells.

Through comprehensive quantitative proteomics comparison of stationary phase/G_0_ and tetrads, we were able to demonstrate that the cellular abundance of proteins in dormant or quiescent cells may be similar and quiescent cells may share similar proteome. For instance, out of 289 common proteins in four biological replicates, only around 40 proteins exhibited fold change ±1.5 out of which only around five proteins possess fold change close to ±4. We identified proteins like Hsp12, Spg4 and Hsp26, which were known to be present in quiescent cells but remain undetected in exponentially growing cells^36^, which further signify sharing of proteome by quiescent cells. Owing to similar abundance of cellular proteins in stationary phase/G_0_ and tetrads, it is anticipated that metabolic profile of these quiescent cells may also be similar. Such future study may provide more mechanistic information about quiescent cells. Thus taken together, previous studies and our present data, it can be concluded that spores can also act as good model for studying properties of stationary phase cells. Further, it is important to mention that we could not detect chromatin proteins in our proteomics investigation. It is known that numerous genes express only during stationary phase (example Hsp26), thus chromatin pull down followed by mass spectrometric analysis may help in identification of novel transcription factors thus helping in understanding the regulation of gene expression in dormant cells.

Our data suggests the importance of Bmh1 and Bmh2 in viability of quiescent cells and cells under stress. We also observed presence of Bmh interacting motifs [RX(1–3)*pS/pT*XP, where X can be any amino acid residue and *pS/pT* represents phosphorylated serine or threonine]^49^ in members of TOR, PKA, PKC and SNF1 pathways (example Byc1, Snf1, Gln3, Msn2), which regulates various aspect of stationary phase or quiescent cells including entering in, viability and exit out of quiescent cells (Table S7). Thus presence of Bmh interacting motifs in members of TOR, PKA, PKC and SNF further highlights the important role of 14-3-3 proteins quiescent stage. Our RT-PCR analysis showed that expression of *BMH1* and *BMH2* is strongly dependent on growth environment as well as stage of cell cycle. Hundreds of proteins are known to interact with budding yeast 14-3-3 proteins, since many of the proteins express or present only in quiescent cells, hence Chip assays or pull down assay of Bmh1/2 from quiescent will further increase the number of Bmh interactors. Any type of such study will further add novel functions and interactors of these conserved proteins known for their diverse roles^50^. Thus it suggests the possibility that like Bmh1/2, human 14-3-3 proteins may also play an important role in the physiology of neuronal cells, which also exits cell cycle and enters into G_0_.

Previous northern blot^36^ and our present RT-PCR data showed complete shutdown of *ACT1* in quiescent cells. While previous studies showed that Actin form stable protein actin bodies, which are highly stable protein structure^51^. These actin bodies provide necessary actin required by stationary phase cells. Further, there is continuous change in the cellular localization of actin in cell while the total level of cellular actin remains almost unchanged as cells passes from active mitotic growth and reaching to the stationary phase^51^. This suggests that there is increased stability of proteins in quiescent cells.

**Figure 7:**
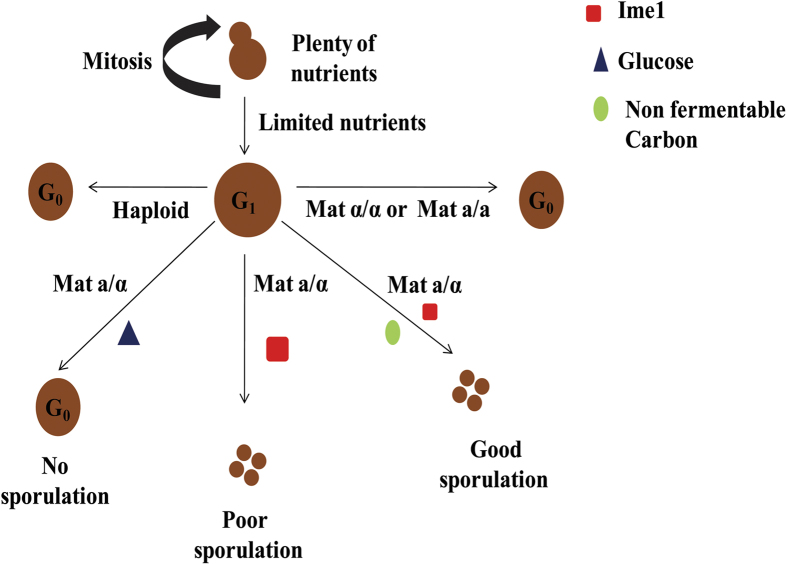
Schematic showing fate of budding yeast cells under nutrient deficiency.

Studies in past have shown that budding yeast cells under absence of essential nutrient(s) like carbon enters dormant state or quiescent state. Even in these dormant state cells remains at G_1_ phase. Haploid budding yeast cells do not express Ime1 in any condition due to Rme1 which prevent expression of Ime1 in haploids. Even diploid cells like Mat a/a or Mat α/α do not express Ime1 due to Rme1. Thus these cells cannot proceed for meiosis and over a time withdraws them from cell cycle. While diploid cells in absence of essential nutrient like carbon or nitrogen also arrests at G_1_ phase. Further, only when other conditions including absence of glucose and presence of non-fermentable carbon (example acetate) is available, cells express Ime1 and embarks for meiosis^29^,^30^,^31^. Thus, under deficiency of essential nutrients cells by default may arrests at G_1_. Nutrients based signalling in yeast is reviewed elsewhere^52^. From our western blot data for Ime1 and previous reports from literature, we propose model for cells under nutrient stress (Fig. 7).

It is known that both tetrads or spores and stationary phase cells share several key features, including low rate of transcription, translation, metabolically less active compared to cycling cells. Our FACS data further showed that in both spores and stationary phase cells remains arrested at G_1_ phase well before START point. Our FACS data and previous reports showed that quiescent cells remains arrested at G_1_ phase^6^,^53^. Apart from that we also showed that just like stationary phase cells, tetrads also retains viability for extended period of time when incubated in sterile water. Stationary phase cells also possess other key properties like accumulation of aminopeptideases in cytoplasm^54^,^55^, characteristically folded chromosomes^56^,^57^, refractive nature^58^, round and numerous mitochondria^59^,^60^. Apart from that there are many properties which also define stationary phase cells^11^. Whether spores or tetrads also share these attributes need to be tested and hence provide opportunities for the contemporary biologists.

To conclude, our proteomic investigation has revealed that the cellular abundance of proteins may be similar in quiescent stages of budding, which includes tetrads or spores and stationary phase/G_0._ Despite low numbers, identification of around 289 (out of more than 5000 protein coding ORFs) common proteins in four biological replicates in present study could be a good point for further studies with emphasis of increasing proteome coverage and greater understanding of quiescent cells. Thus it becomes clear that quiescent cells may share similar proteome both quantitatively as well as qualitatively, except some stage-specific proteins. Further, it will be interesting to compare metabolic profile of tetrads and stationary phase cells, which will certainly broaden our understanding about metabolism of these dormant stages. Further, studies using budding yeast can provide significant insights into such diverse biological processes like pathogenesis, evolution, aging, viability of embryo in seed and long time survival of plant pathogens in crop field(s). Owing to the high degree of conservation of essential proteins from unicellular yeast to metazoans, including humans will certainly help in understanding physiology of neurons, memory B and T cells. Therefore, studying the proteome of stationary cells and tetrads along with multi-omics approaches provides scope for both basic understanding and in applied science.

## Materials and Methods

### Strains, media and culture condition

All the strains used in present study were of SK1 background, unless otherwise mentioned. Diploid wild type strain was used for the stationary phase/G_0_ arrest as well as for sporulation. Primers used in this study are listed in Supp. Table S1. Strains used in this study along with relevant genotypes are listed in Supp. Table S2.

### Induction of stationary phase in budding yeast

Budding yeast cells were arrested at stationary phase as described elsewhere^46^ and briefly explained here. Single colony of diploid strain was inoculated in 5 mL of YPD and tube was incubated at 30 °C, 200 rpm for 24 hour. Twenty four hour grown culture was used to inoculate 50 mL YPD (1% yeast extract, 2% peptone and 2% dextrose) such that initial OD_600nm_ was 0.2. Flask was then incubated at 30 °C; 200 rpm till growth stopped completely by checking OD_600nm_ of culture by spectrophotometer at regular interval of 24 hour (13–14 days after inoculation). Culture was regularly checked for any contamination. Morphology of cells were regularly checked under microscope.

### Alpha factor arrest at G_1_

Alpha factor arrest of required haploid strain was performed as described elsewhere^25^ and briefly described here. Single colony of haploid (Mat a) wild type strain was inoculated in 5 mL YPD it and tube was incubated at 30 °C, 200 rpm for overnight growth. OD_600nm_ of this overnight grown culture was measured and accordingly required volume of cell suspension was transferred to 5 mL YPD such that initial OD_600 nm_ was close to 0.2. 15 μg alpha factor was added to the tube just after setting the OD_600nm_. Tube was incubated at 30 °C, 200 rpm for 4–5 hour. Percentage of G_1_ arrested cells were calculated by counting unbudded and cells with mating projection.

### Thermo-stability assay for stationary cells

Thermal stability or thermal tolerance of G_0_/stationary phase cells was checked as described elsewhere^24^. Known numbers of cells were transferred in 1.5 mL tube and it was incubated for 10 min in water bath maintained at 50 °C. After 10 min, tube was taken out from water bath and immediately transferred into ice bucket for 10 min. Content of tube was plated down on YPD plate along with necessary control. Plates were incubated at 30 °C for 2–3 days. Colonies were counted and percentage of cells that survived heat shock was calculated.

### Induction of meiosis and tetrads formation

Meiotic induction and tetrads formation was performed as described elsewhere^61^ and briefly described here. Initially single colony of diploid wild type strain was inoculated in 5 mL of YPD and tube was incubated at 30 °C, 200 rpm for 24 hour. Same 24 hour grown culture was used to inoculate 50 mL YPA such that initial OD_600nm_ was 0.2. Flask was then incubated at 30 °C, 200 rpm till OD_600nm_ reached to 1.2. Cells were harvested and washed twice in sterile water. Cells were then released in sporulating medium such that OD_600nm_ was 1.0. Flask was incubated at 30 °C, 200 rpm for 24 hour.

### Protein extraction

Protein extraction and quantification was performed as described elsewhere^34^,^62^.

### Buffer exchange, in solution digestion, iTRAQ labelling, LC-MS/MS, Data acquisition and analysis

For quantitative analysis, we labelled protein sample with iTRAQ reagent using 4-plex kit (from AB Sciex). Buffer exchange, in solution digestion, iTRAQ labelling, off gel fractionation, Zip tip and mass spectrometric analysis was performed as described previously. Data acquisition and analysis was performed as explained in detailed elsewhere^63^. MS data (raw files), have been submitted or uploaded to Pride Database (px-submission #83618) (https://www.ebi.ac.uk/pride/archive/).

### Western blot

Western blot and denstiometric analysis for validating proteomics data was performed as described previously^62^.

### Osmotic stress during meiosis

To check the effect of osmotic stress on sporulation, NaCl and KCl was added to sporulation medium at final concentration of 0.3, 0.6, 0.9, 1.2 and 1.5 M.

### Bioinformatics analysis of proteins

Bioinformatics analysis of proteins identified in iTRAQ based quantitative comparison of stationary phase/G_0_ and tetrads were performed as described previously^64^ and also explained here. Proteins which showed common trend in all biological replicates were selected and their Swissprot ID was uploaded on PANTHER software for pathway analysis. Data was exported and PI chart was prepared in MS Excel Sheet.

### cDNA synthesis and RT-PCR

Total RNA isolation, cDNA synthesis and RT-PCR was performed as described previously^62^.

## Additional Information

**How to cite this article**: Kumar, R. and Srivastava, S. Quantitative proteomic comparison of stationary/G_0_ phase cells and tetrads in budding yeast. *Sci. Rep.* **6**, 32031; doi: 10.1038/srep32031 (2016).

## Supplementary Material

Supplementary Information

## Figures and Tables

**Table 1:** Table showing Swissprot ID, protein name and gene symbol for proteins with fold change more than ±1.5 along with fold changes.

Gene symbol^a^	Swissprot ID	Protein name^a^	Fold change^b^
SIP18	P50263.1	Protein SIP18	4.704
RRT5	P43607.1	Regulator of rDNA transcription protein 5	3.071
GAA1	P39012.1	GPI transamidase component GAA1	3.029
HSP12	P22943.1	12 kDa heat shock protein	2.971
GRE1	Q08969.1	Protein GRE1	2.803
ACB1	P31787.3	Acyl-CoA-binding protein	2.747
YCP4	P25349.1	Flavoprotein-like protein YCP4	2.683
ARG1	P22768.3	Argininosuccinate synthase	2.635
POX1	P13711.2	Acyl-coenzyme A oxidase	2.582
PBI1/2	P01095.3	Protease B inhibitor 1	2.591
SPS4	P09937.2	Sporulation-specific protein 4	2.371
RPL28	P02406.3	60S ribosomal protein L28	2.350
CTA1	P15202.1	Peroxisomal catalase A	2.229
SPS100	P13130.1	Sporulation-specific wall maturation protein	2.209
SPG4	Q04438.1	Stationary phase protein 4	2.159
UBI4	P0CG63.1	Polyubiquitin	2.150
FMP45	Q07651.1	SUR7 family protein FMP45	2.125
YBR116C	P38268.1	Putative uncharacterized protein YBR116C	2.106
HTB1	P02293.2	Histone H2B.1	2.080
TEL1	P38110.3	Serine/threonine-protein kinase TEL1	2.040
RPL10	P41805.1	60S ribosomal protein L10	2.037
FOX2	Q02207.1	Peroxisomal hydratase-dehydrogenase-epimerase	1.969
VPS1	P21576.2	Vacuolar protein sorting-associated protein 1	1.941
SOD1	P00445.2	Superoxide dismutase [Cu-Zn]	1.866
HHF1	P02309.2	Histone H4	1.835
HTA1	P04911.2	Histone H2A.1	1.793
CPR1	P14832.3	Peptidyl-prolyl cis-trans isomerase	1.761
EFB1	P32471.4	Elongation factor 1-beta	1.753
TRX2	P22803.3	Thioredoxin-2	1.744
RTC3	P38804.1	Restriction of telomere capping protein 3	1.727
MSH1	P25846.2	DNA mismatch repair protein MSH1, mitochondrial	1.701
RPL5	P26321.4	60S ribosomal protein L5	1.687
MNP1	P53163.1	54S ribosomal protein L12, mitochondrial	1.659
TOM70	P07213.2	Mitochondrial import receptor subunit TOM70	1.644
LSP1	Q12230.1	Sphingolipid long chain base-responsive protein LSP1	1.606
ERG10	P41338.3	Acetyl-CoA acetyltransferas	1.572
THP2	O13539.1	THO complex subunit THP2	1.558
AMS1	P22855.2	Alpha-mannosidase	1.557
CAR2	P07991.2	Ornithine aminotransferase	1.532
RPS7B	P48164.1	40S ribosomal protein S7-B	1.518

All the proteins shown in table showed increased abundance in tetrads.

^a^Gene symbol and protein name as given in uniprot database (http://www.uniprot.org/).

^b^Fold change is given from one set only (although these proteins showed similar trends in all replicates or sets).

We also identified many proteins which showed increased abundance in stationary phase like Hsp26. Data is shown only for proteins showing increased abundance in tetrads.
