# Supplementary material for: Quantitative proteomic comparison of stationary/G0 phase cells and tetrads in budding yeast
Source: Sci Rep. 2016 Aug 25;6:32031. doi: 10.1038/srep32031 (PMC4997312; doi:10.1038/srep32031)
Supplement: Supplementary Information [file srep32031-s1.pdf]

SREP-16-11533A

Supplementary information

Quantitative proteomic comparison of stationary/G_0 phase cells and tetrads in budding yeast

Ravinder Kumar and Sanjeeva Srivastava*

Department of Biosciences and Bioengineering, Indian Institute of Technology Bombay, Powai, Mumbai-400076, India

* Correspondence author:

Dr. Sanjeeva Srivastava, Department of Biosciences and Bioengineering, IIT Bombay, Powai, Mumbai-400 076, India, E-mail: sanjeeva@iitb.ac.in

Phone: +91-22-2576-7779, Fax: +91-22-2572-3480

Legends

Figure S1: Bioinformatics analysis of proteins identified in iTRAQ based quantitative comparison of stationary phase cells and tetrads. (A) Molecular functions and (B) proteins classes.

Figure S2: Cellular abundance of protein in (A) stationary phase and (B) tetrads. (C) Overlap of protein abundance in tetrads and stationary phase cells. (D) Scatter plot showing abundance (average from all replicates from reporter ion intensities) of protein in tetrads (Y-axis) and stationary phase/G_0 cells (X-axis).

Table S1. List of primers used in this study.

Table S2. List of strains used in present study.

Table S3. Parameters used in the analysis of iTRAQ data.

Table S4. List of proteins identified in iTRAQ based proteomics comparison of stationary phase and tetrads.

Table S5. List of ribosomal proteins common in biological replicates along with fold changes.

Table S6: Table showing abundance of proteins in tetrads and stationary phase along with value of t-test.

Table S7: Proteins with Bmh1/2 interacting motifs.

Figure S1

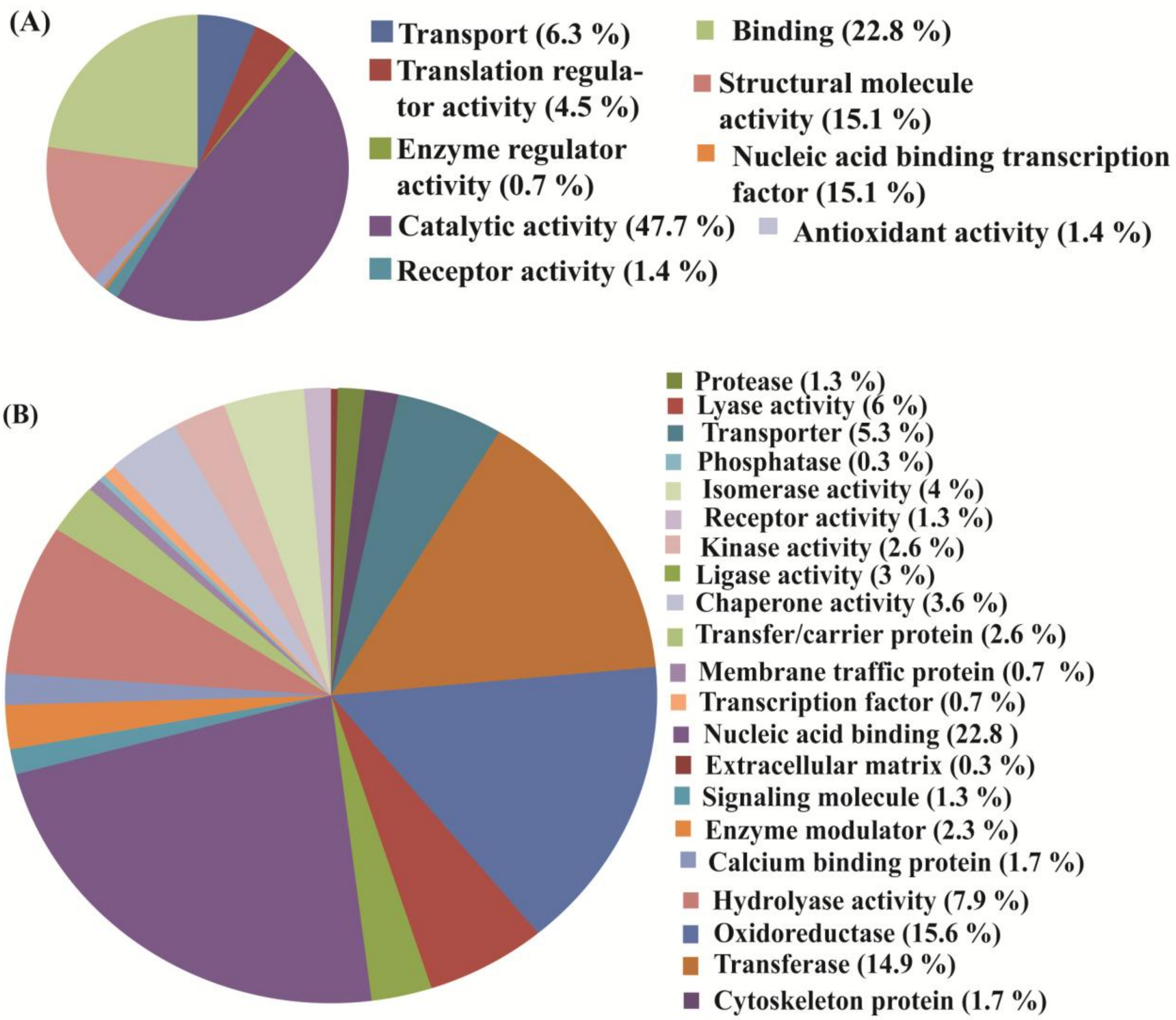
(A)
Transport (6.3 %)
Translation regulator activity (4.5 %)
Enzyme regulator activity (0.7 %)
Catalytic activity (47.7 %)
Receptor activity (1.4 %)
Binding (22.8 %)
Structural molecule activity (15.1 %)
Nucleic acid binding transcription factor (15.1 %)
Antioxidant activity (1.4 %)
(B)
Protease (1.3 %)
Lyase activity (6 %)
Transporter (5.3 %)
Phosphatase (0.3 %)
Isomerase activity (4 %)
Receptor activity (1.3 %)
Kinase activity (2.6 %)
Ligase activity (3 %)
Chaperone activity (3.6 %)
Transfer/carrier protein (2.6 %)
Membrane traffic protein (0.7 %)
Transcription factor (0.7 %)
Nucleic acid binding (22.8)
Extracellular matrix (0.3 %)
Signaling molecule (1.3 %)
Enzyme modulator (2.3 %)
Calcium binding protein (1.7 %)
Hydrolyase activity (7.9 %)
Oxidoreductase (15.6 %)
Transferase (14.9 %)
Cytoskeleton protein (1.7 %)

Figure S2

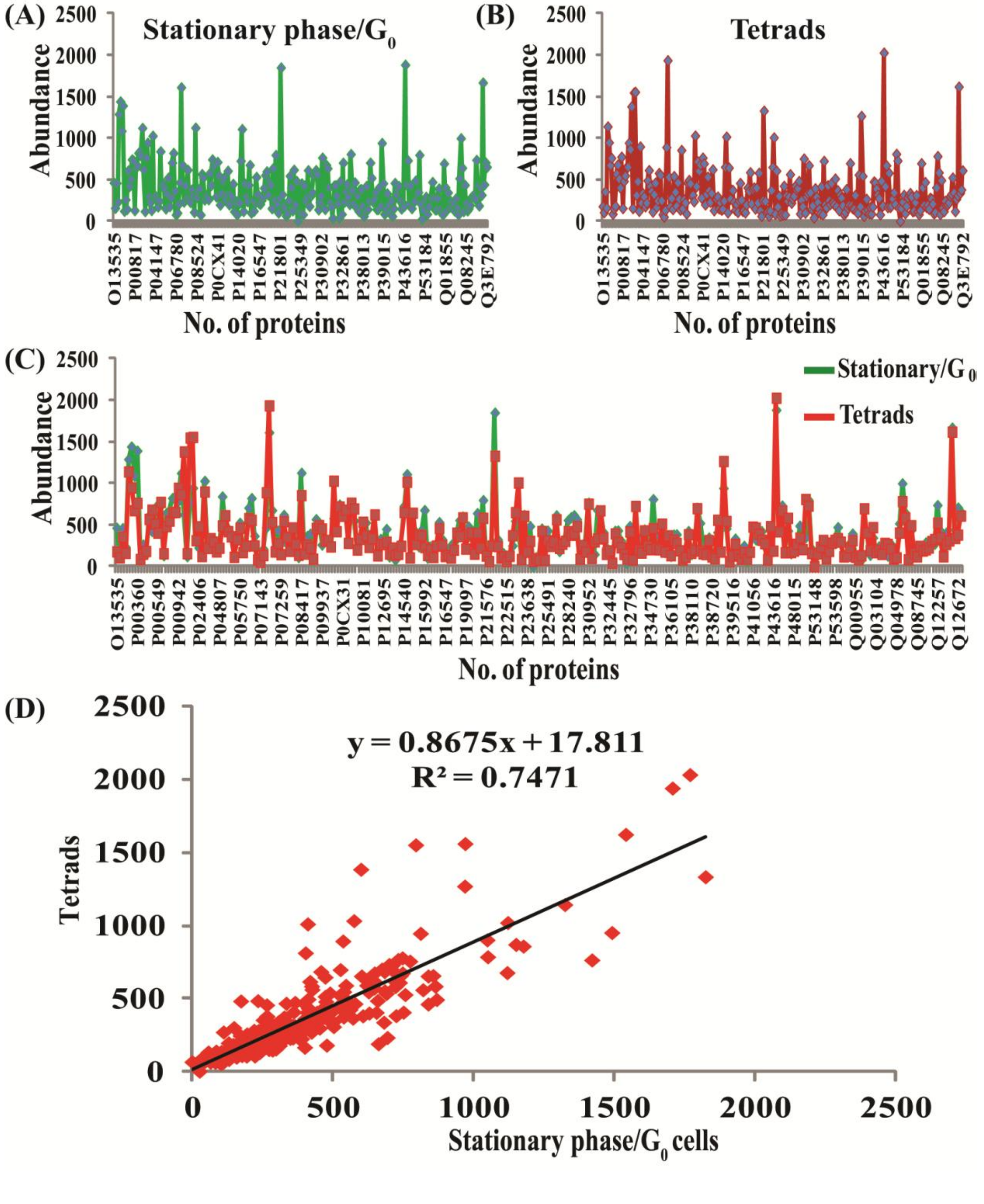
(A)
Stationary phase/G0
Abundance
No. of proteins
(B)
Tetrads
Abundance
No. of proteins
(C)
Stationary/G0
Tetrads
Abundance
No. of proteins
(D)
y = 0.8675x + 17.811
R² = 0.7471
Tetrads
Stationary phase/G0 cells

Table S1.

Name of primer	Description	Nucleotide sequence
RK15	C-terminal tagging of *BMH1*	AACATCAGCAACAGCAGCCACCTGCTGCCGCCGAA GGTGAAGCACCAAAGCGTACGCTGCAGGTCGAC
RK16	C-terminal tagging of *BMH1*	TTTTTTTTCTTTTTTTTTAGTAATTTCTCTTTAGATTTAT CAGAATACTTAATCGATGAATTCGAGCTCG
RK17	Diagnostic for *BMH1* tagging	GGTCAAGCTGAAGACCAA
RK18	Diagnostic for *BMH1* tagging	CTACAAATTATTACACCCCCG
RK23	*BMH1* deletion	CGCAAGCAAGTGAGAAGAAAAAGCAAGTTAAAGATA AACTAAAGATAAAAGCCAGCTGAAGCTTCGTACG
RK24	*BMH1* deletion	CTTTTTTTTTTCTTTTTTTTTAGTAATTTCTCTTTAGATTTA TCAGAATACGGCCACTAGTGGATCTG
RK25	Diagnostic for *BMH1* deletion	CGAGACGAACCGTAACATA
RK26	Diagnostic for *BMH1* deletion	ACACATATAGACATGTACACGC
RK29	*BMH2* deletion	GCCTCTCCCGGTTTTTAATC
RK30	*BMH2* deletion	TCCCCTTGTATTTCTCAGCG
RK47	*BMH2* tagging along with RK30	CTCCTCTTTGGAGGCTTA
RK51	C-terminal tagging of *HSP12*	TCGAATATGTTTCCGGTCGTGTCCACGGTGAAGAAGAC CCAACCAAGAAGCGTACGCTGCAGGTCGAC
RK52	C-terminal tagging of *HSP12*	CACATCATAAAGAAAAAACCATGTAACTACAAAGAGTT CCGAAAGATTTAATCGATGAATTCGAGCTCG
RK53	Diagnostic for *HSP12* tagging	CCACCTCGATTTAAGCGT
RK54	C-terminal tagging of *SPG4*	GACTTTACGAAAGTTTGAGGAGGGGGGAACCCGACAAT AAAGTAAATAGACGTACGCTGCAGGTCGAC
RK55	C-terminal tagging of *SPG4*	GAATAAATAGACAACACAAGAAAAGACACTATGAATATC TCCTCCATTTAATCGATGAATTCGAGCTCG
RK56	Diagnostic for *SPG4* tagging	GGTATTAGTGGCAAAGTTCTG
RK57	*HSP12* deletion	TTCGATAATCTCAAACAAACAACTCAAAACAAAAAAAACT AAATACAACAGCCAGCTGAAGCTTCGTACG
RK58	*HSP12* deletion	TCACACATCATAAAGAAAAAACCATGTAACTACAAAGAGTT CCGAAAGATGGCCACTAGTGGATCTG
RK59	Diagnostic for *HSP12* deletion	GCGTTCTACTTCCTCAATTGCG

Table S2

<u>**Description**</u>	<u>**Genotype**</u>	<u>**Reference**</u>
AMy a/α	Ura^{-}, Leu^{-}, His^{-},Trp^{-}, Kan^{-}	62
AMy α	Ura^{-}, Leu^{-}, His^{-},Trp^{-} , Kan^{-}	62
Mat α *BMH1*-EGFP	Ura^{-}, Leu^{-}, His^{-},Trp^{+}, Kan$^{-,}$	This study
Mat a *BMH1*-EGFP	Ura^{-}, Leu^{-}, His^{-},Trp^{+}, Kan^{-}	This study
Mat a/α *BMH1*-EGFP	Ura^{-}, Leu^{-}, His^{-},Trp^{+}, Kan^{-}	This study
Mat α *BMH2*-EGFP	Ura^{-}, Leu^{-}, His^{-},Trp^{+}, Kan^{-}	This study
Mat a *BMH2*-EGFP	Ura^{-}, Leu^{-}, His^{-},Trp^{+}, Kan^{-}	This study
Mat a/α *BMH2*-EGFP	Ura^{-}, Leu^{-}, His^{-},Trp^{+}, Kan^{-}	This study
Mat a *bmh1*$^{-}$	Ura^{-}, Leu^{-}, His^{-},Trp^{-}, Kan^{+}	62
Mat α *bmh1*$^{-}$	Ura^{-}, Leu^{-}, His^{-},Trp^{-}, Kan^{+}	62
Mat a/α *bmh1*$^{-/-}$	Ura^{-}, Leu^{-}, His^{-},Trp^{-}, Kan^{+}	62
Mat a *bmh2-*	Ura^{-}, Leu^{-}, His^{-},Trp^{-}, Kan^{+}	62
Mat α *bmh2*$^{-}$	Ura^{-}, Leu^{-}, His^{-},Trp^{-}, Kan^{+-}	62
Mat a/α *bmh2*$^{-/-}$	Ura^{-}, Leu^{-}, His^{-},Trp^{-}, Kan^{+}	62
Mat a *HSP12-9myc*	Ura^{-}, Leu^{-}, His^{-},Trp$^{-,}$ Kan^{+}	This study
Mat α *HSP12-9myc*	Ura^{-}, Leu^{-}, His^{-},Trp$^{-,}$ Kan^{+}	This study
Mat a/α *HSP12-9myc*	Ura^{-}, Leu^{-}, His^{-},Trp$^{-,}$ Kan^{+}	This study
Mat a *SPG4-9myc*	Ura^{-}, Leu^{-}, His^{-},Trp$^{-,}$ Kan^{+}	This study
Mat α *SPG4-9myc*	Ura^{-}, Leu^{-}, His^{-},Trp$^{-,}$ Kan^{+}	This study
Mat a/α *SPG4-9myc*	Ura^{-}, Leu^{-}, His^{-},Trp$^{-,}$ Kan^{+}	This study
Mat a *HSP12-GFP*	Ura^{-}, Leu^{-}, His^{+},Trp$^{-,}$ Kan^{-}	This study
Mat a/α *HSP12-GFP*	Ura^{-}, Leu^{-}, His^{+},Trp$^{-,}$ Kan^{-}	This study
Mat a *hsp12*$^{-}$	Ura^{-}, Leu^{-}, His^{-},Trp^{-}, Kan^{+}	This study
Mat α *hsp12*$^{-}$	Ura^{-}, Leu^{-}, His^{-},Trp^{-}, Kan^{+}	This study
Mat a/α *hsp12*$^{-/-}$	Ura^{-}, Leu^{-}, His^{-},Trp^{-}, Kan^{+}	This study
Mat a/α *hsp12*$^{+/-}$	Ura^{-}, Leu^{-}, His^{-},Trp^{-}, Kan^{+}	This study
Mat a/α *IME1-6HA*	Ura^{-}, Leu^{-}, His^{-},Trp^{+}, Kan	This study

Table S3

S. No.	Parameters	Values/condition during analysis
01	SPI	60 %
02	FDR	1 %
03	Species	*S. cerevisiae*
04	No. of mis cleavages	2
05	Enzyme used in protein digestion	Trypsin
06	Data base	SwissProt
07	Variable modification	Oxidation of methionine
08	Fixed modifications	iTRAQ modification at N-terminal and Carbamidomethylation (C)
09	Search engine	Spectrum Mill (Agilent)
10	Pre-fractionation approach used	OFFGEL (Agilent)
11	LC-system	1260 Infinity HPLC-nano-chip
12	Instrument details	6550 Q-TOF iFunnel technology
13	Mass tolerances for MS and MS/MS	20 ppm and 50 ppm
14	Protein pI	3 to 10
15	Batch size	81
16	MS/MS spectral feature filtering	MH+ (400 to 4000 Da)<br>Scan time range (0 to 300 min)<br>Sequence tag length (>1)

Table S4

Swissprot ID	115/114 set 1(fold change*)	117/114 set (fold change*)	Swissprot ID	115/114 set 1(fold change*)	117/114 set(fold change*)	Common proteins
O13535.1	0.404721108	0.395020656	O13535.1	0.364502345	0.383420665	O13535.1
O13539.1	1.558329159	0.666649339	O13539.1	1.137605228	1	O13539.1
P00128.2	0.915733686	0.804966138	P00128.2	0.724973416	0.685866644	P00128.2
P00175.1	0.648869383	0.588453369	P00175.1	0.50662845	0.718470088	P00175.1
P00330.5	1.101141598	1.032398535	P00330.5	1.009051634	1.0181852	P00330.5
P00331.3	0.716480825	0.697855382	P00331.3	0.628506687	0.577142709	P00331.3
P00359.3	0.721464343	0.702222438	P00359.3	0.654742712	0.596254436	P00359.3
P00360.3	0.615999037	0.579949827	P00360.3	0.495171436	0.51370072	P00360.3
P00401.2	0.780786493	0.505926601	P00401.2	0.655651007	0.885153765	P00401.2
P00410.1	0.892546971	0.475329551	P00410.1	0.804408371	0.629378587	P00410.1
P00424.1	1.029540083	1.28788163	P00424.1	1.186736798	1.948008537	P00424.1
P00427.1	0.96727633	0.86154616	P00427.1	0.962594443	0.985549337	P00427.1
P00445.2	1.866065983	2.286276671	P00445.2	1.519924856	1.04608494	P00445.2
P00447.1	1.236275261	1.043188594	P00447.1	1.154285418	0.941478465	P00447.1
P00549.2	0.615145672	0.568408487	P00549.2	0.549808075	0.518350551	P00549.2
P00560.2	1.251796459	1.130530567	P00560.2	1.108800644	1.085981856	P00560.2
P00817.4	1.197478705	1.241427492	P00817.4	1.326845141	1.591072968	P00817.4
P00830.2	0.844400887	0.791685866	P00830.2	0.711038705	0.676424116	P00830.2
P00890.2	0.893785162	0.826450318	P00890.2	0.813379198	0.713507253	P00890.2
P00924.3	0.886996305	0.852634892	P00924.3	0.803293997	0.762072415	P00924.3
P00925.2	0.847919965	0.824733549	P00925.2	0.784584098	0.729004689	P00925.2
P00942.2	1.41029796	1.267512522	P00942.2	1.215036792	1.099616149	P00942.2
P00950.3	0.904379378	0.869947353	P00950.3	0.785672517	0.712518807	P00950.3
P01095.3	2.51925996	2.337554497	P01095.3	4.544117059	3.033535091	P01095.3
P01123.2	0.867538687	0.879649076	P01123.2	2.881859498	1.22010051	P01123.2
P02293.2	2.080600533	1.954771533	P02293.2	2.302178983	2.385017745	P02293.2
P02309.2	1.835279765	1.879045498	P02309.2	1.971098674	1.729074463	P02309.2
P02400.2	1.209994089	1.054091423	P02400.2	0.912565489	1.106497353	P02400.2
P02406.3	2.350552657	1.547564994	P02406.3	2.772139771	1.359428242	P02406.3
P02992.1	0.379981214	1.058484395	P02992.1	0.847919965	0.723969086	P02992.1
P02994.1	1.054091423	0.944747041	P02994.1	0.862741345	0.775393206	P02994.1
P04037.1	0.757333158	0.777546036	P04037.1	0.597495602	0.89564567	P04037.1
P04147.4	1.350037985	1.230291345	P04147.4	1.594384953	1.151089491	P04147.4
P04456.4	1.491744027	1.173648178	P04456.4	1.168777249	1.101905116	P04456.4
P04806.2	1.4063932	1.017479692	P04806.2	0.927230546	0.815637493	P04806.2
P04807.4	1.029540083	0.582366793	P04807.4	1.126619228	0.558643569	P04807.4
P04840.4	0.662503509	0.622005827	P04840.4	0.645281245	0.542614686	P04840.4
P04911.2	1.793776319	1.571345033	P04911.2	2.034959384	1.781385801	P04911.2
P05317.2	1.114193651	0.980099415	P05317.2	1.121943481	1.012554807	P05317.2
P05319.1	1.101141598	1.034547582	P05319.1	0.796640096	0.89564567	P05319.1

P05626.2	0.684441907	1.631274987	P05626.2	0.462011286	0.610896551	P05626.2
P05694.4	1.185092771	1.095052471	P05694.4	0.815072332	0.949342121	P05694.4
P05750.5	1.093535457	0.988970916	P05750.5	1.017479692	0.920187651	P05750.5
P05755.4	1.129747215	1.086734863	P05755.4	1.051172909	0.86934456	P05755.4
P05756.3	1.45296505	0.644387315	P05756.3	0.771640088	0.631563631	P05756.3
P06168.1	0.997922719	0.877821798	P06168.1	0.867538687	0.754712984	P06168.1
P06169.7	0.736113431	0.677832163	P06169.7	0.693034943	0.611320139	P06169.7
P06208.1	0.793883931	0.616853585	P06208.1	0.608361179	0.571569168	P06208.1
P06780.3	1.110338834	0.782411782	P06780.3	1.112650121	4.386286354	P06780.3
P07143.1	0.718968266	0.715984371	P07143.1	0.456915725	0.306508715	P07143.1
P07149.2	0.647970483	0.545631939	P07149.2	0.809442217	0.691595315	P07149.2
P07213.2	1.644900137	2.193143177	P07213.2	1.620006947	1.857033705	P07213.2
P07246.2	1.244011653	1.36983298	P07246.2	1.491744027	1.343503426	P07246.2
P07251.5	0.898132373	0.86934456	P07251.5	0.789493887	0.811689581	P07251.5
P07256.1	0.76684133	0.611744021	P07256.1	0.62981499	0.71449707	P07256.1
P07257.1	0.664342907	0.637280314	P07257.1	0.751059963	0.613867842	P07257.1
P07259.5	0.44534645	0.483973513	P07259.5	0.561749952	0.578344092	P07259.5
P07275.2	1.391846392	1.355664327	P07275.2	0.635956503	0.605416542	P07275.2
P07280.2	1.309485423	1.397646972	P07280.2	1.041021598	1.391846392	P07280.2
P07342.1	0.898755127	0.76418826	P07342.1	1.079228237	0.754712984	P07342.1
P07991.2	1.53261996	1.326845141	P07991.2	1.374588696	1.470187336	P07991.2
P08004.2	1.260503392	1.124278924	P08004.2	1.360370852	0.830470024	P08004.2
P08067.1	0.668037039	0.863339559	P08067.1	1.137605228	1.264879542	P08067.1
P08417.2	0.87175824	0.852634892	P08417.2	0.682073917	0.73153561	P08417.2
P08524.2	0.635075491	0.42720487	P08524.2	0.554784736	0.654289036	P08524.2
P08679.1	0.819036698	0.708578698	P08679.1	0.430474594	0.600818025	P08679.1
P09201.2	0.624598063	0.667111585	P09201.2	0.520150133	0.4248425	P09201.2
P09232.1	1.461044379	2.165950091	P09232.1	0.957935218	0.323985241	P09232.1
P09457.1	0.947370071	0.865136691	P09457.1	0.867538687	0.897510051	P09457.1
P09624.1	1.107264584	0.925946023	P09624.1	0.986916546	0.966606097	P09624.1
P09937.2	2.371828999	2.100889088	P09937.2	2.09216988	2.00416321	P09937.2
P0C0W1.2	1.016070143	0.676424116	P0C0W1.2	1.081474763	1.203303026	P0C0W1.2
P0C2H6.1	1.478362431	0.920825697	P0C2H6.1	1.051172909	0.582366793	P0C2H6.1
P0C2H9.1	1.099616149	1.0238469	P0C2H9.1	1.020304659	0.939522749	P0C2H9.1
P0CG63.1	2.150988781	1.660940048	P0CG63.1	1.652900636	1.657489809	P0CG63.1
P0CS90.1	1.343503426	1.300440147	P0CS90.1	0.971980988	0.853226098	P0CS90.1
P0CX23.1	0.981459064	1.118061851	P0CX23.1	0.928516852	0.837987135	P0CX23.1
P0CX31.1	1.280759861	1.035982764	P0CX31.1	1.090507733	1.069547088	P0CX31.1
P0CX35.1	1.111879158	1.012554807	P0CX35.1	1.065846736	0.787307977	P0CX35.1
P0CX37.1	1.194163187	1.043188594	P0CX37.1	1.030968319	0.891928519	P0CX37.1
P0CX41.1	1.286989247	1.202469249	P0CX41.1	1.411275843	1.202469249	P0CX41.1
P0CX45.1	1.4054187	1.478362431	P0CX45.1	1.418140036	1.186736798	P0CX45.1
P0CX53.1	1.068805991	1.148698355	P0CX53.1	1.035982764	0.89688816	P0CX53.1
P0CX55.1	1.176906737	1.222640278	P0CX55.1	0.577142709	0.919550046	P0CX55.1

P10081.3	1.328685814	1.159899655	P10081.3	1.122721422	1.094293701	P10081.3
P10591.4	0.782411782	0.670821112	P10591.4	0.693515485	0.660669203	P10591.4
P10622.3	1.058484395	0.820741609	P10622.3	0.712518807	0.85027416	P10622.3
P10664.4	1.265756594	0.96727633	P10664.4	1.140763716	1.033114388	P10664.4
P10963.2	1.333298677	1.135242102	P10963.2	1.016774673	0.91319825	P10963.2
P11154.2	0.729004689	0.666649339	P11154.2	0.462011286	0.662503509	P11154.2
P11484.3	0.875998315	0.79940583	P11484.3	0.801625329	0.686342216	P11484.3
P12695.1	0.820172911	0.907519155	P12695.1	0.915733686	0.807201075	P12695.1
P12709.3	0.695923196	0.631563631	P12709.3	0.62546454	0.664803554	P12709.3
P13130.1	2.20992897	1.582274602	P13130.1	2.168954818	2.593679109	P13130.1
P13663.1	1.011853201	0.792234811	P13663.1	1.038139271	1.136816973	P13663.1
P13711.2	2.582914701	1.364147835	P13711.2	4.086880331	2.73966596	P13711.2
P14020.3	0.877213549	0.657015814	P14020.3	0.753667455	0.672217497	P14020.3
P14127.1	1.350974085	1.107264584	P14127.1	1.068805991	1.169587664	P14127.1
P14540.3	1.139973273	1.068805991	P14540.3	0.997231251	0.901875378	P14540.3
P14681.1	0.982139595	0.830470024	P14681.1	0.971980988	1.110338834	P14681.1
P14742.4	1.099616149	1.031683179	P14742.4	2.164449289	0.591725511	P14742.4
P14832.3	1.761738748	1.695839929	P14832.3	1.5888688	1.271913007	P14832.3
P15108.4	1.04608494	0.965936329	P15108.4	1.089752112	0.968618189	P15108.4
P15202.1	2.229932437	2.298989696	P15202.1	1.911890635	1.797510253	P15202.1
P15703.1	0.918912883	1.024556823	P15703.1	0.721464343	0.811127156	P15703.1
P15992.3	0.364249778	0.299992846	P15992.3	0.263340259	0.263888429	P15992.3
P16120.1	1.284315809	1.286097483	P16120.1	1.902636553	1.821339667	P16120.1
P16140.2	0.724973416	0.948684315	P16140.2	1.586667686	0.653382627	P16140.2
P16387.2	1.034547582	0.589678296	P16387.2	0.584793832	0.792234811	P16387.2
P16451.1	1.29056249	1.502119927	P16451.1	1.759298152	2.856007959	P16451.1
P16474.1	0.851453708	1.071030823	P16474.1	0.746389192	0.757333158	P16474.1
P16521.4	1.048989328	0.845572287	P16521.4	0.955282936	0.485653748	P16521.4
P16547.2	1.016774673	0.87175824	P16547.2	0.484644908	0.221902834	P16547.2
P16861.1	0.721964598	0.666649339	P16861.1	1.016774673	0.555939579	P16861.1
P16862.4	0.345318612	0.360482218	P16862.4	0.516915368	1	P16862.4
P17255.3	0.716480825	0.804408371	P17255.3	0.982139595	0.574747424	P17255.3
P17505.2	1.202469249	1.071030823	P17505.2	1.027401439	0.993092495	P17505.2
P17709.1	0.749499801	0.660669203	P17709.1	0.642157904	0.583579051	P17709.1
P18239.2	1.047536127	1.008352455	P18239.2	0.969289817	0.923382311	P18239.2
P19097.2	0.710546022	0.596254436	P19097.2	0.750019495	0.643940815	P19097.2
P19262.2	0.808320869	0.81056512	P19262.2	0.892546971	0.946057647	P19262.2
P19414.2	1.071030823	1.027401439	P19414.2	0.961927455	0.912565489	P19414.2
P19657.3	1.155085785	1.167967395	P19657.3	0.948684315	0.759962428	P19657.3
P19882.1	0.722465199	0.640823962	P19882.1	0.618566239	0.542238704	P19882.1
P20967.2	0.944092419	0.915099168	P20967.2	0.901875378	0.881480158	P20967.2
P21538.2	0.847332435	0.876605721	P21538.2	0.824162085	0.76154437	P21538.2
P21576.2	1.94126894	0.912565489	P21576.2	0.360982299	0.380244688	P21576.2
P21657.3	0.784040454	0.869947353	P21657.3	0.317317722	0.394200087	P21657.3

P21801.1	0.890692901	0.815072332	P21801.1	1.284315809	1.234562607	P21801.1
P22082.1	0.784040454	0.788946841	P22082.1	0.655196702	0.701735863	P22082.1
P22133.2	0.729004689	0.662044455	P22133.2	0.971307496	0.685391402	P22133.2
P22203.4	0.615999037	0.424253951	P22203.4	0.411510173	0.489710149	P22203.4
P22219.4	1.336074078	1.202469249	P22219.4	0.758383773	0.624165274	P22219.4
P22515.2	0.711038705	0.589269704	P22515.2	0.640379931	0.562919293	P22515.2
P22768.3	2.653690281	1.293248932	P22768.3	1.196648963	0.732042848	P22768.3
P22803.3	1.744725412	2.11696879	P22803.3	2.556198726	2.060507907	P22803.3
P22855.2	1.557249382	1.082224645	P22855.2	1.491744027	1.214194884	P22855.2
P22943.1	2.971105841	3.08656035	P22943.1	3.127478573	3.482202253	P22943.1
P23254.4	0.317978252	0.302918165	P23254.4	0.647970483	0.780786493	P23254.4
P23301.3	1.217566019	1.170398641	P23301.3	1.033830736	1.071030823	P23301.3
P23638.1	1.104198847	1.002081605	P23638.1	1.466116757	0.692074858	P23638.1
P23641.1	0.641712949	0.706127202	P23641.1	0.564482202	0.688725023	P23641.1
P23644.1	1	1	P23644.1	1	1	P23644.1
P25087.4	0.981459064	1.151887642	P25087.4	1.293248932	0.789493887	P25087.4
P25349.1	2.68328445	0.726482525	P25349.1	0.350624813	1	P25349.1
P25443.3	1.246601194	1.175276328	P25443.3	0.930449658	0.834509281	P25443.3
P25451.1	0.679714121	0.590087172	P25451.1	0.536258308	0.715984371	P25451.1
P25491.1	0.918276162	0.630251696	P25491.1	1	0.388773018	P25491.1
P25613.1	1.32592576	1.036701101	P25613.1	1.150291893	0.975355462	P25613.1
P25694.3	1.355664327	1.088997015	P25694.3	0.639049682	0.754712984	P25694.3
P25846.2	1.701727459	0.706616822	P25846.2	0.957271458	0.419284092	P25846.2
P26321.4	1.687631592	1.099616149	P26321.4	0.989656656	1.254402205	P26321.4
P26783.3	0.97063447	1.216722359	P26783.3	0.798298386	0.933032992	P26783.3
P26786.4	0.854409741	0.885767519	P26786.4	1.092020546	0.924022572	P26786.4
P28240.1	0.853226098	0.750019495	P28240.1	0.783497187	0.677362489	P28240.1
P28241.1	0.765248385	0.71946679	P28241.1	0.670821112	0.616853585	P28241.1
P28834.2	0.68491649	0.659753955	P28834.2	0.628942486	0.619424349	P28834.2
P29453.3	1.043911927	0.90062598	P29453.3	0.568802614	0.636838738	P29453.3
P29547.2	0.920187651	0.194251817	P29547.2	0.650220073	0.266646445	P29547.2
P30624.1	0.791685866	1.244011653	P30624.1	1.871246996	1.946658748	P30624.1
P30902.2	0.579547976	0.458819941	P30902.2	0.458502022	0.512633619	P30902.2
P30952.1	1.163120042	1.057750964	P30952.1	1.047536127	0.942131274	P30952.1
P31539.2	0.939522749	0.637280314	P31539.2	0.484309095	0.720964436	P31539.2
P31787.3	2.747272467	3.056753042	P31787.3	1.710004356	1.665551542	P31787.3
P32191.2	0.585605091	0.458184322	P32191.2	0.578344092	0.461691155	P32191.2
P32316.2	1.269270886	1.169587664	P32316.2	1.128182137	1.017479692	P32316.2
P32324.1	0.990342872	0.941478465	P32324.1	0.901875378	0.924663278	P32324.1
P32327.2	0.957271458	0.662044455	P32327.2	0.462011286	0.446273486	P32327.2
P32445.1	1.379360922	1.218410264	P32445.1	1.666706414	1.054822317	P32445.1
P32463.1	0.41754396	0.774319028	P32463.1	1.417157397	1.586667686	P32463.1
P32471.4	1.753211443	1.463071221	P32471.4	0.928516852	0.886996305	P32471.4
P32473.2	0.768970416	0.76154437	P32473.2	0.647072827	0.580754366	P32473.2

P32582.1	0.695440986	0.562139462	P32582.1	0.557869661	0.774319028	P32582.1
P32589.4	0.874784765	0.704172113	P32589.4	0.532923368	0.608783009	P32589.4
P32603.1	1	1	P32603.1	2.515769944	7.361501205	P32603.1
P32796.2	1.093535457	1.117287138	P32796.2	0.846745312	0.738669032	P32796.2
P32861.1	1.084477409	0.963261894	P32861.1	0.817902059	0.930449658	P32861.1
P32905.3	1.313121125	1.131314463	P32905.3	0.948684315	0.980779004	P32905.3
P33303.2	0.418703244	0.50382688	P33303.2	1.112650121	2.115501927	P33303.2
P33327.1	0.674551267	0.531816336	P33327.1	0.481964904	0.431370672	P33327.1
P33442.2	1.148698355	1.027401439	P33442.2	1.038139271	1.00765376	P33442.2
P34227.1	1.010451446	0.898132373	P34227.1	0.986916546	0.583983697	P34227.1
P34730.3	0.948684315	1.016070143	P34730.3	1.286989247	0.620283649	P34730.3
P34760.3	0.706616822	0.677832163	P34760.3	0.53329289	0.513344773	P34760.3
P35169.3	1.327765158	1.503161478	P35169.3	1	1	P35169.3
P35691.1	1.426025717	1.154285418	P35691.1	1.601029621	0.883315051	P35691.1
P36008.1	0.989656656	1.00486382	P36008.1	1.119612889	1.067325338	P36008.1
P36010.1	0.968618189	0.800514811	P36010.1	0.933032992	0.832198735	P36010.1
P36060.1	1.301341855	1.385109468	P36060.1	1.014662547	0.996540263	P36060.1
P36105.1	1.251796459	0.862143545	P36105.1	1.237132479	0.925304428	P36105.1
P37291.2	1.091263877	0.992404375	P37291.2	0.916368645	0.67877249	P37291.2
P37292.2	0.7944344	0.853226098	P37292.2	0.689680461	0.608361179	P37292.2
P38013.4	1.373636233	1.053361036	P38013.4	1.227735684	1.130530567	P38013.4
P38067.2	0.785672517	1.014662547	P38067.2	1	1	P38067.2
P38071.3	1.208317843	1.158292806	P38071.3	1.054091423	1.237990291	P38071.3
P38077.1	1.003471749	0.933032992	P38077.1	0.827596816	0.890075733	P38077.1
P38110.3	2.040609318	1.294145654	P38110.3	0.791685866	1.00695555	P38110.3
P38111.1	1.286097483	0.890692901	P38111.1	0.581560021	0.62546454	P38111.1
P38149.1	1.132883885	1.051901779	P38149.1	1.476314406	0.908148418	P38149.1
P38268.1	2.106722072	1.55293775	P38268.1	0.325110037	0.300409012	P38268.1
P38325.1	0.524494664	0.751059963	P38325.1	1.04608494	0.808320869	P38325.1
P38695.1	0.584388624	0.384751805	P38695.1	0.570777354	0.532185091	P38695.1
P38701.3	1.059952783	0.983502074	P38701.3	0.826450318	0.937571096	P38701.3
P38720.1	0.647521499	0.659296807	P38720.1	0.60332196	1.059952783	P38720.1
P38788.2	0.881480158	0.839731493	P38788.2	0.770037174	0.85027416	P38788.2
P38804.1	1.727876375	1.592176198	P38804.1	1.685293659	1.370782805	P38804.1
P38891.1	0.93109482	0.62981499	P38891.1	1.095811766	1.150291893	P38891.1
P39012.1	3.029332632	0.949342121	P39012.1	1.453972517	0.515126977	P39012.1
P39015.3	1.510472586	1.45195828	P39015.3	1.576800348	1.646040691	P39015.3
P39109.2	0.617709319	0.797192477	P39109.2	1	1	P39109.2
P39516.2	1.417157397	0.942131274	P39516.2	1.109569472	1.071773463	P39516.2
P39522.2	1.208317843	0.851453708	P39522.2	0.812815602	0.642603169	P39522.2
P39676.2	0.584388624	0.6341957	P39676.2	0.57236208	0.97063447	P39676.2
P39726.3	0.568408487	0.333324669	P39726.3	1	3.144869169	P39726.3
P39954.1	0.69495911	0.468136124	P39954.1	0.72597914	0.537374586	P39954.1
P39990.1	1.072516617	1.312211255	P39990.1	2.061936638	2.973165969	P39990.1

P40582.1	1.204972315	1.479387509	P40582.1	1.0181852	1.025978145	P40582.1
P41056.2	1.256142381	1.132098902	P41056.2	1.30224419	1.050444544	P41056.2
P41338.3	1.572434584	1.678299274	P41338.3	1.837825767	1.362258035	P41338.3
P41805.1	2.037782393	1.199139914	P41805.1	1.130530567	0.97063447	P41805.1
P41939.2	0.919550046	0.855002178	P41939.2	0.791137301	0.632878297	P41939.2
P41940.2	1.41029796	0.940826108	P41940.2	1.630144665	1.110338834	P41940.2
P42222.2	0.481297222	0.657927263	P42222.2	0.459138081	0.326012684	P42222.2
P43607.1	3.071620539	2.526254524	P43607.1	2.229932437	1.864772973	P43607.1
P43616.1	1.472226862	1.146312186	P43616.1	0.994470169	0.994470169	P43616.1
P43620.1	1.22010051	1.181811547	P43620.1	1.225185332	1.029540083	P43620.1
P43635.1	1.121166078	0.912565489	P43635.1	1.028113827	0.833931044	P43635.1
P46367.2	1.051901779	1	P46367.2	0.927873476	0.89564567	P46367.2
P46655.3	0.930449658	0.683020128	P46655.3	0.871154192	0.767905135	P46655.3
P47139.2	1.470187336	1.207480591	P47139.2	1.136029265	1.607701981	P47139.2
P47176.1	0.224688782	0.656560563	P47176.1	1.890804234	1.624504793	P47176.1
P48015.2	0.615572207	0.723969086	P48015.2	0.790589117	0.687770909	P48015.2
P48164.1	1.51887169	1.215036792	P48164.1	1.003471749	1.278099363	P48164.1
P48415.2	0.813379198	0.638164384	P48415.2	0.581560021	0.679243142	P48415.2
P50107.1	0.722465199	0.55632506	P50107.1	0.742261785	0.50768305	P50107.1
P50263.1	4.704365001	4.236873339	P50263.1	2.969047141	2.012515647	P50263.1
P52593.1	1.057750964	0.679714121	P52593.1	1.353786279	1.172022284	P52593.1
P53090.1	0.787853886	0.447202451	P53090.1	1	1.304954948	P53090.1
P53148.1	1	1	P53148.1	1	1	P53148.1
P53163.1	1.659789171	1.250062303	P53163.1	0.727490342	0.893785162	P53163.1
P53184.1	1.189207115	0.965267025	P53184.1	0.926588062	0.814507563	P53184.1
P53221.2	1.422077411	1.436940177	P53221.2	1.189207115	1.073260286	P53221.2
P53228.1	0.86154616	1	P53228.1	2.711328654	0.736623843	P53228.1
P53252.1	1.465100875	1.268391399	P53252.1	1.41029796	1.154285418	P53252.1
P53312.1	1.053361036	0.90312651	P53312.1	1.0132569	1.092020546	P53312.1
P53598.1	1.129747215	1.171210181	P53598.1	1.163926534	0.956608158	P53598.1
P54115.4	0.85086373	0.670821112	P54115.4	0.735603373	0.768970416	P54115.4
P54839.1	0.768970416	0.916368645	P54839.1	1.594384953	1.411275843	P54839.1
P81449.2	1.199139914	1.272794935	P81449.2	1.118837101	0.252088114	P81449.2
P81451.1	1.081474763	0.917639882	P81451.1	1.165541198	1.755643595	P81451.1
Q00055.4	0.821310701	0.911933166	Q00055.4	0.984866443	0.85027416	Q00055.4
Q00711.1	0.961927455	0.944747041	Q00711.1	0.84264683	0.918912883	Q00711.1
Q00955.2	0.582770599	0.679714121	Q00955.2	0.986232704	1.494849249	Q00955.2
Q01477.1	1.094293701	0.607097442	Q01477.1	0.31930344	1	Q01477.1
Q01560.1	0.641712949	1.362258035	Q01560.1	1	1	Q01560.1
Q01574.2	1.17772279	1.063632673	Q01574.2	1.088242442	1.038859103	Q01574.2
Q01855.1	0.910669834	1.026689546	Q01855.1	1.312211255	1.159095952	Q01855.1
Q02207.1	1.969732886	1.542210825	Q02207.1	1	1	Q02207.1
Q02486.1	1.465100875	1.398616083	Q02486.1	1.682958965	1.777685362	Q02486.1
Q03104.1	1.104198847	1.446934886	Q03104.1	0.634635443	1.101141598	Q03104.1

Q03161.1	1.202469249	1.047536127	Q03161.1	0.579146403	0.743291492	Q03161.1
Q03937.1	1.136816973	1.297738767	Q03937.1	1.231144413	1.56049096	Q03937.1
Q04409.1	1.294145654	1.209994089	Q04409.1	1.117287138	1.104198847	Q04409.1
Q04438.1	2.15995312	2.251677172	Q04438.1	1.872544495	3.525920632	Q04438.1
Q04792.1	1.111108729	1.021012126	Q04792.1	0.955945318	0.854409741	Q04792.1
Q04947.1	0.333324669	0.259175275	Q04947.1	1.226884977	0.788400174	Q04947.1
Q04978.1	1.42899414	1.196648963	Q04978.1	5.101777567	1.641483218	Q04978.1
Q06405.1	1.118837101	1.033830736	Q06405.1	0.592135806	0.626332219	Q06405.1
Q06679.1	1.033114388	0.781869643	Q06679.1	1.094293701	1.227735684	Q06679.1
Q07478.1	0.78024548	0.763129604	Q07478.1	1.747145792	1.36983298	Q07478.1
Q07500.1	1	0.352086057	Q07500.1	2.774061938	0.881480158	Q07500.1
Q07651.1	2.125791349	1.872544495	Q07651.1	1	1	Q07651.1
Q08245.3	0.938871747	0.837987135	Q08245.3	0.586824089	0.847919965	Q08245.3
Q08745.1	0.677832163	0.647072827	Q08745.1	1.062159186	0.771640088	Q08745.1
Q08969.1	2.803054898	2.698205069	Q08969.1	1	1	Q08969.1
Q12031.1	1.104964485	1.123499903	Q12031.1	0.681129017	0.716977624	Q12031.1
Q12165.1	0.764718139	0.752623374	Q12165.1	0.816203046	0.558256481	Q12165.1
Q12207.1	1.196648963	0.903752727	Q12207.1	1.002776436	0.412652704	Q12207.1
Q12213.3	0.972654947	0.803293997	Q12213.3	1.151887642	1.180992661	Q12213.3
Q12230.1	1.606587994	1.504203751	Q12230.1	1.385109468	1.350037985	Q12230.1
Q12257.1	0.788946841	0.754712984	Q12257.1	0.607939642	0.657927263	Q12257.1
Q12289.1	0.934327347	0.910038824	Q12289.1	0.833931044	0.778085177	Q12289.1
Q12305.1	0.942131274	0.762600827	Q12305.1	0.584793832	0.423372656	Q12305.1
Q12335.1	0.701735863	0.538866573	Q12335.1	0.463294031	0.459138081	Q12335.1
Q12349.1	0.922103118	0.72597914	Q12349.1	1.152686347	0.942784536	Q12349.1
Q12420.1	1.062895674	1.164733586	Q12420.1	0.813943185	0.814507563	Q12420.1
Q12428.1	0.796640096	0.778624691	Q12428.1	0.885153765	0.840313752	Q12428.1
Q12672.1	0.616853585	0.782954296	Q12672.1	0.76418826	0.519429552	Q12672.1
Q3E792.1	1.513616793	1.260503392	Q3E792.1	0.890075733	0.708578698	Q3E792.1
O13516.3	1.549711862	0.790589117	P01094.1	2.561519723	1.304050735	
O14455.3	1.811267966	1.53581027	P02829.1	1.092777739	1.016774673	
P00045.1	0.883927531	0.853817714	P06738.4	2.032140286	1.536875181	
P00358.3	0.704172113	0.666649339	P07170.2	0.547146851	0.823020345	
P00931.1	1.272794935	2.27205853	P07267.1	1.320422841	0.807760778	
P04449.1	1.846764621	1.793776319	P0CX82.1	1.948008537	2	
P04802.3	2.064797071	3.391679859	P0CX84.1	1.278985581	2.45206972	
P05318.4	0.418413121	1.043911927	P11076.3	1.009051634	0.885153765	
P05739.4	1.009051634	0.647072827	P25303.2	1	1	
P06105.3	1.135242102	0.625031151	P25356.2	0.768970416	0.859160755	
P06777.1	1	1.473247686	P30771.1	0.536258308	0.804408371	
P06843.1	1.707635429	0.957935218	P32333.1	0.736113431	0.7031966	
P07283.1	0.765778999	1	P35497.1	0.643494624	0.989656656	
P07284.2	1.318593614	1.445932295	P36126.3	4.597979392	3.40109664	
P07703.1	0.813943185	0.798298386	P38334.1	0.213750544	0.53998828	

P08468.2	2.158456473	2.924114897	P38800.1	0.401925495	0.303759198	
P08518.2	0.829319546	0.579146403	P38840.3	0.532923368	1.284315809	
P08638.1	1.168777249	0.959929261	P38879.3	0.76154437	0.87539133	
P0CX39.1	0.712025098	0.515126977	P40185.1	0.825877665	0.297095776	
P0CX49.1	1.837825767	0.808881348	P40413.3	0.673616788	0.683493726	
P10592.3	0.784040454	0.679243142	P40433.1	0.616426163	0.406407801	
P15019.4	0.651573575	0.76154437	P40471.1	0.705637922	0.71548826	
P16892.2	0.894404902	0.762072415	P46672.2	1.114193651	1.481439798	
P17695.3	0.988970916	0.923382311	P47127.1	1.090507733	0.775393206	
P19146.3	1.0453601	0.86934456	P53130.1	0.365261095	0.71400199	
P19955.1	1.010451446	0.633756261	P53982.1	0.518350551	0.652929894	
P21242.2	0.61813763	1.418140036	Q02326.2	1.587767862	1.129747215	
P21691.2	1.434949535	1.260503392	Q03558.3	1.125058485	0.492774668	
P21954.1	0.56097174	0.632878297	Q03654.1	0.591725511	0.739181216	
P22137.1	1.092777739	0.871154192	Q05016.1	1	1	
P22147.1	2.326240083	2.150988781	Q06681.1	1.322254605	0.931740429	
P23542.3	0.742261785	0.469761375	Q12089.1	0.441045683	0.781869643	
P23639.1	0.730522189	0.366529189	Q12675.1	0.51584159	0.903752727	
P25294.1	1	1	Q12680.2	1.048989328	0.963261894	
P25339.2	1.215879283	1.095052471	Q99326.1	1.252664439	1.117287138	
P26637.1	1	1				
P26785.3	1.336074078	1.22858698				
P27796.1	1	1				
P28272.1	0.295862755	0.272438028				
P28625.2	0.905006463	0.791137301				
P31116.1	1.20664392	1.686462221				
P32264.2	0.855595026	0.651573575				
P32336.2	0.535144349	0.984866443				
P32386.2	0.780786493	0.475659138				
P32802.2	1	1				
P33331.2	0.651573575	0.373712312				
P33416.2	0.2913853	0.648869383				
P35179.2	1.581178233	1.041743429				
P36002.3	0.750019495	0.848507902				
P36091.1	1.348167732	2.108182847				
P36224.2	0.774319028	0.35404386				
P37012.1	2.080600533	1.496922987				
P37296.2	0.512633619	2.100889088				
P38115.1	1	1				
P38219.1	1.236275261	1.035982764				
P38756.1	0.690637224	0.565657231				
P38806.1	2.615342697	1.025978145				
P39526.1	1.976571303	0.886996305				
P39925.1	1.89605393	4.41373496				

P39935.2	1.760518027	0.846745312				
P39958.1	1.070288698	1.280759861				
P40053.1	0.898132373	0.847332435				
P40075.3	1.53261996	1.785093943				
P40468.1	1.830198336	1.812523877				
P40486.1	0.93109482	0.677362489				
P40513.1	1	1				
P41911.2	1.937236378	1				
P47029.2	1.258757174	0.5090926				
P47061.1	0.256672386	0.406971593				
P47068.2	1.0181852	0.601234624				
P47084.2	1.29056249	1.04608494				
P48239.1	1.448942155	0.811127156				
P48526.2	1	1				
P50113.1	1.246601194	0.978063473				
P51401.1	2.383365149	2.140577397				
P52910.1	1.065846736	0.791685866				
P53091.2	0.853817714	0.901250463				
P53188.1	3.164549205	2.687006851				
P53272.1	3.133988748	7.066520412				
P53540.1	0.580351957	0.571965487				
P53877.1	1.919858522	1.25092908				
P53978.2	0.97874165	0.973329374				
P54000.1	1.185914499	1.365093718				
P54113.1	0.914465089	1.486582984				
P54114.1	1.445932295	1.151887642				
P80210.3	1.542210825	0.482968164				
Q01532.3	1.151887642	1.937236378				
Q02196.1	0.995159722	1.039579435				
Q02206.2	0.200267469	0.406971593				
Q03028.1	2.039195366	1.173648178				
Q03102.1	1	0.797192477				
Q03193.1	0.71548826	0.918912883				
Q03195.1	0.949342121	1.186736798				
Q03280.1	2.989698497	3.912253894				
Q04458.1	1	1				
Q05022.1	1.305859787	1.074004472				
Q05498.1	1.20664392	0.425431865				
Q05946.1	1	0.666649339				
Q05948.1	0.848507902	1.132883885				
Q06151.1	2.433444717	1.547564994				
Q06321.1	0.821310701	1.0453601				
Q06494.1	1	1				
Q06624.1	1.454980684	1				

Q07629.1	1	2.901904416				
Q08204.1	1.561572985	1.531557997				
Q08921.1	1.192508872	0.759962428				
Q12009.1	0.888226796	0.577943353				
Q12032.1	0.317537746	2.515769944				
Q12159.2	3.331103084	2.799171731				
Q12181.1	0.586417475	0.579949827				
Q12283.2	1.115739322	1				
Q12303.1	0.492433221	0.682546859				
Q12329.1	1	0.450000965				
Q12690.1	1.901318202	2.136130816				
Q3E754.1	1.395710764	1.051901779				
Q96VH5.1	0.550570799	0.590905773				

*Fold change (converted log2ratio of iTRAQ115/114, 117/114 as given in protein summary file exported from spectrum mill from Agilent)

Table S5

RPP2B	P02400.2	1.209994	1.054091	0.912565	1.106497
RPL28	P02406.3	2.350553	1.547565	2.77214	1.359428
RPL25	P04456.4	1.491744	1.173648	1.168777	1.101905
RPP0	P05317.2	1.114194	0.980099	1.121943	1.012555
RPP2A	P05319.1	1.101142	1.034548	0.79664	0.895646
RPL6B	P05739.4	1.009052	0.647073	1	1
RPS3	P05750.5	1.093535	0.988971	1.01748	0.920188
RPS13	P05756.3	1.452965	0.644387	0.77164	0.631564
RPS19A	P07280.2	1.309485	1.397647	1.041022	1.391846
RPS22A	P0C0W1.2	1.01607	0.676424	1.081475	1.203303
RPL27A	P0C2H6.1	1.478362	0.920826	1.051173	0.582367
RPL31B	P0C2H9.1	1.099616	1.023847	1.020305	0.939523
RPL20A	P0CX23.1	0.981459	1.118062	0.928517	0.837987
RPS24A	P0CX31.1	1.28076	1.035983	1.090508	1.069547
RPS4A	P0CX35.1	1.111879	1.012555	1.065847	0.787308
RPS6A	P0CX37.1	1.194163	1.043189	1.030968	0.891929
RPL23A	P0CX41.1	1.286989	1.202469	1.411276	1.202469
RPL2A	P0CX45.1	1.405419	1.478362	1.41814	1.186737
RPL12A	P0CX53.1	1.068806	1.148698	1.035983	0.896888
RPS18A	P0CX55.1	1.176907	1.22264	0.577143	0.91955
RPP1B	P10622.3	1.058484	0.820742	0.712519	0.850274
RPL4A	P10664.4	1.265757	0.967276	1.140764	1.033114
RPS17B	P14127.1	1.350974	1.107265	1.068806	1.169588
RPS2	P25443.3	1.246601	1.175276	0.93045	0.834509
RPL5	P26321.4	1.687632	1.099616	0.989657	1.254402
RPS5	P26783.3	0.970634	1.216722	0.798298	0.933033
RPS7A	P26786.4	0.85441	0.885768	1.092021	0.924023
RPL8B	P29453.3	1.043912	0.900626	0.568803	0.636839
RPS0A	P32905.3	1.313121	1.131314	0.948684	0.980779
RPS1A	P33442.2	1.148698	1.027401	1.038139	1.007654
RPL14A	P36105.1	1.251796	0.862144	1.237132	0.925304
RPS20	P38701.3	1.059953	0.983502	0.82645	0.937571
RPS14B	P39516.2	1.417157	0.942131	1.109569	1.071773
RPL33B	P41056.2	1.256142	1.132099	1.302244	1.050445
RPL10	P41805.1	2.037782	1.19914	1.130531	0.970634
RPS7B	P48164.1	1.518872	1.215037	1.003472	1.278099
RPL26B	P53221.2	1.422077	1.43694	1.189207	1.07326
RPS15	Q01855.1	0.91067	1.02669	1.312211	1.159096
RPS10A	Q08745.1	0.677832	0.647073	1.062159	0.77164
RPL7B	Q12213.3	0.972655	0.803294	1.151888	1.180993

RPL21B	Q12672.1	0.616854	0.782954	0.764188	0.51943
RPS25A	Q3E792.1	1.513617	1.260503	0.890076	0.708579

*****Gene symbol was taken from uniprot (http://www.uniprot.org/uniprot)

Table 6

Swissprot ID	iTRAQ114[a]	iTRAQ114[a]	iTRAQ116[a]	iTRAQ116[a]	iTRAQ115[a]	iTRAQ117[a]	iTRAQ115[a]	iTRAQ117[a]	t-test[b]
O13535	413.4	482.7647059	488.2051282	463.9411765	184.9487179	180.4722222	171.75	173.8125	0.000342978
O13539	102	80	390	60	159	68	91	NA	0.565144241
P00128	398.5483871	445.047619	482.9263158	509.4888889	383.7282609	323.0879121	377.1363636	339.8139535	0.014946526
P00175	225.6521739	205.4444444	216.16	282.625	176.0526316	158.8095238	125.75	181.875	0.017263249
P00330	1179.97654	1251.992481	1357.136628	1365.076923	1211.795918	1123.083095	1112.431818	1108.381679	0.034693144
P00331	1283.51875	1490.026316	1471.400621	1514.681034	963.1206349	929.0444444	956.6902655	942.2857143	0.002293544
P00359	985.8909091	1054.408163	1174.853854	1129.420918	704.2289528	680.2127216	669.2663185	641.3994709	0.001168871
P00360	1299.52901	1305.880952	1539.131313	1418.377953	813.432526	788.4666667	722.936	716.7083333	0.000476614
P00401	172.8	61	152.6666667	103	145.5	74.4	56	71	0.312055643
P00410	306	180.8333333	310	244	219.6666667	146.3333333	130.8	106	0.032587916
P00424	142.6666667	129.3333333	161.6666667	145.6666667	189	207.6666667	184	149.6666667	0.044218499
P00427	495.5454545	642.6	603.3636364	694.6	495.9090909	518.1	642	617.2	0.491287484
P00445	316.2	567	408.8	398	693.75	572	862	593	0.023941114
P00447	431.875	553.4166667	466.7878788	571.6666667	514.3030303	474.125	537.0833333	518.6666667	0.894682835
P00549	715.894382	688.0760234	859.0527523	706.0526316	438.3920188	432.8588808	384.8427673	357.4294479	0.001034047
P00560	672.1334842	692.1521739	794.0890869	755.5245902	832.4166667	751.8404494	786.5138122	727.3405405	0.252893784
P00817	113.7142857	106.4705882	117.4	184	129.7818182	139.3043478	181	171.2666667	0.302042918
P00830	594.6802974	641.2897196	682.3457249	654.0599078	493.054717	472.7307692	497.647619	466.1658537	0.001229052
P00890	625.9403409	637.8356164	738.1823204	677.5973154	648.8806818	502.460452	591.5454545	451.9375	0.065343183
P00924	765.6410256	780.754386	883.6110325	853.4280702	692.3052326	640.9035088	652.0320285	616.1956522	0.003976488
P00925	795.5222222	786.31	923.5798898	859.5016835	694.8397163	665.7818697	642.7938144	608.2	0.004211497
P00942	706.9202899	777.8484848	828.6304348	828.6461538	939.056338	882.2898551	1001.515625	946.578125	0.006329915
P00950	1033.755853	1063.709402	1224.668896	1164.333333	915.3892617	864.451505	850.1452991	834.8290598	0.006195141
P01095	691.75	300	949	551	1630.75	1617	1363	910	0.013744587
P01123	68.45454545	79.5	118.7142857	208	82.25	56.16666667	340	144	0.630688944
P02293	671.6111111	622.7222222	900.3207547	861.7222222	1613.603774	1522.166667	1576.055556	1476.444444	0.000412262
P02309	860.1428571	824.75	1087.285714	998.75	1652.571429	1568.571429	1677.5	1323.5	0.001198802

P02400	337.7027027	232.0909091	330.4047619	273.9090909	403.7560976	324.2142857	230.9090909	294.1	0.669624261
P02406	371	114	257	148	872	574	316	155	0.202984754
P02992	201.6666667	98.5	136	91	87.33333333	256	78.5	66.5	0.858237406
P02994	961.4427083	1023.00625	1106.490862	1017.006135	975.4882507	903.4713542	873.6125	838.4968944	0.021239935
P04037	234	328.8333333	279.4545455	318.5	174.4545455	162.5	237	304.1666667	0.12805594
P04147	297.7272727	241.1818182	361.6818182	315.5454545	376.7083333	324.3913043	332.1818182	314.0909091	0.304645757
P04456	274.5128205	203.4615385	344.6052632	205.8461538	402.5405405	321.0512821	231.8461538	225.4615385	0.505254124
P04806	181	170.05	179.25	170.3333333	209.7931034	165.3103448	176	157.625	0.874251708
P04807	206.3333333	238.7368421	256.3636364	264.3	305.98	118.3877551	325.4705882	136.2777778	0.744222334
P04840	750.4639175	869.6724138	854.8172414	883.2393162	498.9964664	464.6210526	502.9826087	491.0521739	0.000760381
P04911	456.1818182	356.1333333	529.3823529	371.1764706	728.6857143	664.2857143	544.4444444	514.3888889	0.03018399
P05317	413.7567568	394.5	485.6929825	505.7380952	449.1681416	411.2857143	424.4186047	397.7272727	0.373039165
P05319	378.1875	364.8571429	456.8297872	381.6190476	407.28	399.3333333	307.9047619	322.5	0.317842974
P05626	109	217.5	132.25	143.25	107.8	106	88.5	148.25	0.219201098
P05694	277.4516129	335.125	336.4193548	352.25	352.3666667	321.483871	300.3125	316.25	0.895845639
P05750	503.2698413	478.86	586.5813953	520.38	522.1181102	479.144	439.3396226	401.2777778	0.126260626
P05755	146.3157895	178.7777778	152.8	237.2222222	177.9473684	143.4210526	204.7777778	149.6666667	0.710227154
P05756	231.3333333	243	373	288	325.3333333	161	269	232	0.462240913
P06168	647.2055556	688.3417722	746.5	733.4177215	635.6648045	581.4858757	560.8481013	536.6835443	0.006812029
P06169	819.1658768	739.0828402	950.4095238	772.2209302	650.0780856	579.3052109	528.6049383	467.1282051	0.005105172
P06208	341.483871	400	397.9393939	324	283.1	231.6969697	240.8571429	239.7692308	0.003795498
P06780	73.33333333	52	64	121	63.5	74.14285714	49	136	0.903967997
P07143	94.26666667	88.85714286	114.9090909	101.4285714	56.08333333	65.63636364	44.2	41.8	0.000909915
P07149	225.4042553	157.8823529	261.2346939	189.1212121	158.5731707	140.2413793	119.0689655	118.2758621	0.03697255
P07213	257.6666667	611	409	588	689.4	573.1666667	1095	1202	0.062936665
P07246	1320.026316	1549.25	1753.641026	1819.4375	1943.307692	1959.205128	1922.125	1916	0.062875455
P07251	626.634375	625.4566929	776.9588608	669.1875	579.0383387	556.4089457	481.34375	509.2479339	0.01892015
P07256	297.5151515	145.8823529	322.3548387	169.2857143	243.0645161	195	143.9375	122.5	0.319330711
P07257	424.8105263	385.7857143	508.3555556	397	319.3033708	279.5494505	275.8918919	236.8205128	0.005662565
P07259	246.739	242	283.840	330.166	133.129	131.851	171.133	140.176	0.0036

	1304		5797	6667	0323	8519	3333	4706	79552
P07275	589.5	621	977	283	760	635	395	376	0.6725 58818
P07280	328.272 7273	193.75	337.909 0909	227.75	442.545 4545	427.909 0909	228.75	305	0.2577 53897
P07342	208.694 9153	215.578 9474	252.696 4286	240.882 3529	205.396 2264	179.980 3922	188.222 2222	164.5	0.0164 06287
P07991	328.220 339	349.619 0476	449.175 4386	391.857 1429	501.209 6774	431.983 871	434.545 4545	496.523 8095	0.0425 56933
P08004	192.333 3333	289	224.933 3333	499.5	212.066 6667	189.5	414.5	259.5	0.7180 35908
P08067	88.5	167	68.3333 3333	112.5	56	75.25	265	152.333 3333	0.6169 74642
P08417	968.526 7857	1089.75	1194.27 027	1247.5	876.302 7523	843.100 9174	867.222 2222	833.043 4783	0.0204 33863
P08524	270.117 6471	291.666 6667	312.882 3529	287.833 3333	192.75	113.882 3529	144.666 6667	155	0.0009 32467
P08679	322.333 3333	429.727 2727	360.916 6667	341.454 5455	234.192 3077	268.608 6957	281.545 4545	256.9	0.0148 7497
P09201	421.064 5161	316.384 6154	503.166 6667	322.461 5385	310.482 7586	272.645 1613	166.25	140.166 6667	0.0321 15611
P09232	67.75	71	96.4285 7143	73.5	110.333 3333	126.428 5714	73.5	50.5	0.5207 54407
P09457	525.858 5859	519.326 087	629.242 4242	589.680 8511	482.939 3939	482.577 3196	445.510 6383	432.152 1739	0.0199 51885
P09624	472.242 4242	493.526 3158	517.235 2941	622.157 8947	543.272 7273	434.333 3333	486.428 5714	514.210 5263	0.4669 57403
P09937	240.942 8571	223.406 25	308.726 0274	264.333 3333	518.556 962	460.737 5	406.909 0909	426.303 0303	0.0009 71841
P0C0W1	390	295.6	264.25	292.8	351.25	267.25	322.8	282.8	0.8945 51453
P0C2H6	333.75	239	405	214.5	481.75	367.75	250.5	162	0.8405 22582
P0C2H9	245.947 3684	264.857 1429	264.35	272.285 7143	248.222 2222	214.3	268.285 7143	219.571 4286	0.1521 71946
P0CG63	582.529 4118	475.933 3333	757.647 0588	492.333 3333	1300.73 5294	1151.27 2727	846	817.266 6667	0.0225 31088
P0CS90	346.684 2105	545.333 3333	438.333 3333	404.166 6667	438.263 1579	372.684 2105	448.333 3333	422.333 3333	0.7836 74824
P0CX23	855.066 6667	593.333 3333	925.133 3333	596.333 3333	891.933 3333	916.785 7143	556.888 8889	504.666 6667	0.8637 39359
P0CX31	402	687.5	657.666 6667	866.5	519.5	401.333 3333	752.5	751	0.7276 30824
P0CX35	674.616 6667	620.103 4483	776.508 1967	654.379 3103	760.245 9016	721.573 7705	672.724 1379	567.689 6552	0.9879 64834
P0CX37	287.513 5135	288.090 9091	335.425	270	310.875	286.947 3684	286.428 5714	239.5	0.5110 76464
P0CX41	650.260 8696	680.142 8571	843.521 7391	672.142 8571	833.086 9565	720.913 0435	799.857 1429	698.428 5714	0.3859 85471
P0CX45	616.96	328.444 4444	781.76	469.888 8889	949.92	863.52	489.888 8889	472	0.3968 93746
P0CX53	232.8	163.230 7692	241.227 2727	153.384 6154	257.695 6522	243.857 1429	156.153 8462	143.818 1818	0.9442 19689
P0CX55	307.347 8261	321.5	421.521 7391	285	372.826 087	398.391 3043	231	311.5	0.9139 26061
P10081	451.888 8889	427.75	526.657 8947	515.062 5	549.487 1795	518.513 5135	546.533 3333	521.933 3333	0.1073 5146
P10591	485.747 4048	527.586 2069	547.277 027	540.213 6752	397.736 4621	348.398 5765	390.333 3333	343.883 9286	0.0002 15707

P10622	268.25	257.4	303.55	234.9	306.65	253.4210526	187	180.2	0.3567229
P10664	324.6481481	278.52	393.0566038	273.1538462	404.1538462	339.82	292.2692308	308.92	0.628402884
P10963	523.8333333	611.7288136	672.1612903	615.7741935	724.3899371	578.8774194	655.5625	529.5	0.769721419
P11154	178.7272727	198	215.2727273	206.6	145.9642857	144.2692308	110.7142857	117.8	0.001175439
P11484	331.1925134	320.7571429	359.2879581	300.3648649	306.7472527	277.7032967	261.3	217.0666667	0.037626181
P12695	345.5714286	224.3333333	377.5952381	223.4285714	331.7804878	328.1463415	227.3846154	186.4285714	0.670741106
P12709	434.4722222	419.76	500.6698113	438.3673469	334.6767677	293.592233	273	276.3913043	0.000643475
P13130	66.5	59	145	195	168	155.5	128	153	0.37018783
P13663	280	218.5	251.8888889	140.6666667	267.2222222	248.8888889	178.3333333	203.6666667	0.963096688
P13711	67.66666667	23	147	NA	285.5	105.75	94	63	0.394269714
P14020	177.5	268.5	205	345	154	103.25	174	161	0.066471178
P14127	188.0769231	245.2	258.9166667	192.8	265.7692308	206.6923077	247	260	0.335989692
P14540	684.1847826	672.56	789.9893617	757.472973	684.0952381	626.3709677	695.3157895	604.4933333	0.089887076
P14681	1064.076923	1069.4	1076.230769	1221	957.8461538	964.5384615	1054.6	1086.6	0.115834372
P14742	179.2222222	97.75	139.8333333	62.375	138.1428571	141.5	79	60.28571429	0.661812971
P14832	394.1126761	464.9655172	481.375	472.2068966	668.1388889	659.75	640.2413793	603.4482759	0.000393657
P15108	275.3859649	281.88	300.5491803	313.212766	285.4358974	250.3589744	279.8541667	252.8958333	0.086704754
P15202	158.3333333	180	275.6666667	341	377.8888889	404.7777778	383.75	340.75	0.042191554
P15703	448.4	371.3333333	537.4666667	389	414.6666667	445.4	302.3333333	329.6666667	0.256115441
P15992	619.8917197	691.3859649	710.6184211	679.7818182	242.1567164	239.744	232.5319149	206.2608696	2.88825E-05
P16120	92	81.66666667	131	98.66666667	119	124.6666667	154	139.3333333	0.048329117
P16140	208.5862069	222.0833333	249.2857143	267.5833333	189.9259259	199.8571429	240.4	181.6363636	0.117972041
P16387	144.5	159	128.5	60.5	147.25	103.6666667	62.5	126	0.656592808
P16451	243.6363636	85	343.4166667	198.5	277.5454545	283.7	192	236.5	0.632978167
P16474	513.0769231	457.3333333	678.0769231	476	524.0769231	530.3076923	418.3333333	359	0.307445807
P16521	276.7692308	328.3846154	371.5128205	321.2142857	317.4210526	225.2894737	293.3333333	192.3846154	0.109418205
P16547	191.4	229.3333333	263.25	183.6666667	168.6	150	100.5	68.33333333	0.018970782
P16861	234	96.66666667	309.4736842	185	189.7058824	174.4375	98.66666667	66.5	0.2235374
P16862	333	147	373	148	115	120	76	NA	0.088939092
P17255	269.906	274	333.285	286	222.95	207.025	220.421	144.05	0.0087

	9767		7143			641	0526		55718
P17505	370.303 5714	378.711 8644	427.244 898	421.041 3223	437.954 386	393.303 8869	388.280 9917	392.429 8246	0.8506 96259
P17709	503.952 381	518.62	629.380 9524	565.823 5294	384.717 1717	376.576 087	360.166 6667	329.041 6667	0.0031 26695
P18239	527.104 2945	601.797 4684	618.585 3659	643.687 5	583	554.006 2893	625	587.632 9114	0.7355 44543
P19097	299.052 6316	327.906 25	311.568 9655	331.729 7297	238.52	196.849 0566	209.676 4706	198.941 1765	0.0001 67486
P19262	664.882 3529	289.8	710.882 3529	247.181 8182	559.294 1176	525.470 5882	225.818 1818	227.818 1818	0.5629 24945
P19414	412.5	437.258 6207	455.215 9091	437.779 661	423.555 5556	408.047 619	404.245 9016	376	0.0489 48536
P19657	127.909 0909	185.307 6923	191.612 9032	111.736 8421	168.193 5484	153.5	139.833 3333	139.687 5	0.8661 37909
P19882	592.603 5503	649.223 8806	693	621.656 7164	434.751 4793	389.25	403.640 625	379.954 5455	0.0002 72661
P20967	240.769 2308	235.119 0476	288.586 7769	258.454 5455	235.289 7196	225.846 8468	222.815 7895	202.307 6923	0.0592 62677
P21538	591	797	747	1049	494	566.5	657	607	0.1048 67803
P21576	110	313	211.333 3333	194	171.666 6667	103.666 6667	113	119	0.1487 43713
P21657	78	104	94.5	132	71.1666 6667	98.25	33	41	0.0730 76419
P21801	269.833 3333	242.222 2222	341.958 3333	268.222 2222	254.916 6667	262.958 3333	257.111 1111	217.888 8889	0.2401 73618
P22082	1923	1685.83 3333	2077.58 3333	1710.66 6667	1509.91 6667	1493.33 3333	1137.33 3333	1174.66 6667	0.0089 45039
P22133	300.538 4615	291.72	351.714 2857	341.115 3846	251.538 4615	221.571 4286	267.826 087	227.095 2381	0.0060 54638
P22203	162.25	210.333 3333	106.166 6667	264	101.375	79.5	102	129	0.0868 71731
P22219	104.666 6667	199.5	165.25	142	129	77.8571 4286	137.75	130.25	0.2142 86503
P22515	63.5	70.8	79.8333 3333	43.5	71.8	52.6666 6667	74	47.75	0.7890 2955
P22768	77.5	111.4	119.230 7692	74.8	169.625	123.5	130.428 5714	75	0.2572 88146
P22803	194.25	224.333 3333	293.111 1111	280.333 3333	328.555 5556	359.333 3333	472	329	0.0275 66218
P22855	377	594	484	729	587	408	886	721	0.4422 28279
P22943	394.435 7143	337.578 9474	505.978 7234	388.2	1112.30 5556	1077.89 6552	958.868 8525	878.796 875	0.0001 97523
P23254	225	90.3333 3333	117.6	80.6666 6667	94.25	85.25	73.3333 3333	88.3333 3333	0.2848 18643
P23301	515.431 8182	624.05	693.790 6977	640.333 3333	615.136 3636	612.681 8182	593.809 5238	603.047 619	0.7664 26372
P23638	163.75	209.857 1429	185.2	233.857 1429	175.4	141.2	235.857 1429	140.142 8571	0.3962 09334
P23641	422	468.909 0909	527.264 7059	512.916 6667	307.424 2424	299.545 4545	321.8	289.416 6667	0.0032 46871
P23644	NA	NA	NA	NA	81	NA	NA	47	#DIV/0 !
P25087	97.6470 5882	108.875	104.166 6667	122.875	88.6	108.789 4737	112.8	72.6666 6667	0.2938 39862
P25349	95	77	98.5	NA	150	50.5	27	NA	0.7421 89867

P25443	296.1052632	579.7333333	392.5789474	547.0714286	335.7894737	342.8888889	518.7857143	496.9333333	0.727602904
P25451	93.5	79.66666667	98.71428571	52	84.5	77	46	103	0.839671226
P25491	521.5	265	559	366	523.5	345.75	265	103	0.329069082
P25613	222.4545455	252.4583333	222.6551724	289.4583333	269.3965517	222.122807	246.6666667	236.0416667	0.871159288
P25694	235.5	340.5	263.7	342	319.7	331.2222222	246.5	276	0.952859673
P25846	536.2	775	633.8	505	802	385.6	742	325	0.735752561
P26321	180.7704918	168.8636364	203.3934426	168.0526316	262.9491525	207.7230769	202.5833333	205.4583333	0.066691961
P26783	282.4642857	264.9411765	354.2083333	320.5384615	271.9285714	349.9090909	216.4444444	241.2222222	0.354706721
P26786	293.1891892	395.125	319.7647059	371.8235294	277.3333333	274.0571429	350.3529412	312.7777778	0.21275406
P28240	535.8023599	511.4353741	639.2514793	533.8053691	471.9546828	432.4096386	418.6142857	363	0.011505875
P28241	542.647541	594.7288136	611.1666667	615.0847458	405.4132231	364.15	394.8793103	348.8813559	7.98303E-05
P28834	563.807377	624.1188119	642.8852459	620.8823529	406.5	383.7905983	422.6276596	374.8613861	0.000113208
P29453	619.2857143	501	896	242	708.5714286	613.8571429	285	319	0.647646097
P29547	175	180	90	113	161	34	117	48	0.236577653
P30624	323.4	93	346.6	145	270.8	389.6	174	181	0.752031
P30902	280.4761905	301.8571429	332.35	295.5	195.5789474	205.6666667	134.7142857	164	0.001002764
P30952	733.8411552	686.4803922	908.4578755	727.8365385	857.5842294	779.9751773	704.3809524	666.8942308	0.860154556
P31539	198.8	88	232.1428571	144	143.1666667	118.6	64.66666667	70.5	0.132472884
P31787	132.3055556	159.1	161.3030303	125.3333333	312.175	327.9736842	283.3846154	267.0769231	0.00019331
P32191	657.2542373	653.5862069	736.2068966	654.6896552	378.0172414	341.0877193	329.7586207	289.4137931	1.73958E-05
P32316	600.1921569	585.2830189	719.0742188	640.6320755	742.1215686	676.3961538	645.8867925	620.3490566	0.415514135
P32324	327.8863636	437.6129032	366.3076923	506.9354839	312.9230769	296.0333333	390.28125	380.71875	0.220958282
P32327	193.6551724	220	246.7037037	238.375	180	164.9130435	124	121.5	0.007038274
P32445	133.55	143.4285714	176.4736842	226	185.1052632	179.3529412	213.5714286	178.1428571	0.441645501
P32463	51.71428571	26.5	35.83333333	29	43.4	36.2	48	43	0.325879349
P32471	265.7142857	572.3	283.8823529	582.9090909	358.5882353	294.8235294	492.1818182	433	0.761208659
P32473	408.1875	427.5333333	416.8787879	468.2857143	321.8333333	300.4666667	267.3333333	280.7142857	0.000263281
P32582	158.6666667	527	230.3333333	316	111.6666667	93	294	408	0.487172736
P32589	197.92	204.5555556	246.1538462	194.3333333	174	134.12	137.6666667	141	0.006316291
P32603	NA	33	NA	41	72	NA	60	145	0.1700

									72984
P32796	454.4210526	467.0952381	510.4358974	499.5714286	445.2307692	455.5384615	382.9047619	387.2380952	0.034568606
P32861	93	73.33333333	81	92	59	112.5	63.33333333	82.5	0.695810992
P32905	680.1363636	624.8333333	826.9090909	680.3333333	852.8636364	795.3181818	610.6666667	638.6666667	0.781490853
P33303	331.8	110.5	352.8	136	263.2	209.6	112.5	214.5	0.665801737
P33327	211.0909091	475.5	263.8181818	461.25	142.6363636	104.2	214	193.75	0.061476627
P33442	522.0888889	316.9230769	578.0444444	347.5	592.7272727	533.6136364	284.6428571	309.8461538	0.917417084
P34227	306.6976744	257.5	340.2093023	262.9090909	290.9069767	271.0232558	256.2	184.9090909	0.224386176
P34730	258.2307692	128.4	276.3333333	153.5	280.3333333	270.2	137.5	128.8	0.998723334
P34760	712.1958763	778.7647059	864.3092784	873.9411765	494.6701031	454.5102041	472.4242424	415.7941176	0.001012393
P35169	356.75	NA	389	NA	330.8333333	292.6	43	NA	0.234546311
P35691	175.4736842	190.625	214.1578947	262.1428571	218.2777778	219	216	154.375	0.737438864
P36008	444.8461538	473.5714286	473.0769231	472.7857143	480	483.9166667	581.4166667	501.4285714	0.148382054
P36010	222.8	139.25	199	116.75	227.7	211.7	150	113	0.871814625
P36060	258	344.3636364	323.9666667	312.7	348.59375	370.125	367.8333333	393.5454545	0.0383534
P36105	128.3333333	109.1818182	137.9545455	110.6666667	139.6190476	120.9	138.5	124.2	0.321827805
P37291	374.4705882	356.2941176	382.475	410.3125	326.4390244	361.7714286	313.5555556	274.7777778	0.032912276
P37292	329.5555556	466.625	307.2777778	429	227	275.1111111	301	316.125	0.068015274
P38013	186.8	156.625	175	211.5	217.5555556	196.2	180.375	166.5	0.646073701
P38067	95.5	NA	41.75	43	70.5	137	NA	29	0.637747601
P38071	103.7777778	119	127.25	155.6666667	100.4	103.5	174	60	0.549293328
P38077	411.5203252	400.15	474.3306452	406.0714286	420.1826087	407.5701754	345.3488372	363.8	0.167883687
P38110	122	201.5	114.8	138	192.2	149.3333333	132	167	0.524949115
P38111	220	237.5	325	184	283	196	137.5	161.5	0.32257474
P38149	791	445	1027	551	896	832	657	404	0.97193133
P38268	94	646	280	1060	198	146	210	194	0.21657784
P38325	263.125	180.9	273	219.8888889	116	161.5	179.375	160.375	0.023904637
P38695	216.1	310	251.6	298	133.9	106.3333333	177	165	0.004620331
P38701	304.4852941	358.5217391	380.2253521	338.2692308	345.3125	304.8	311.16	293.75	0.163331299
P38720	147.2307692	156	140.6470588	130.7777778	108.6153846	102.625	112.1111111	142.8333333	0.049537858

P38788	108.8333333	100	156.8	217	104.75	116.8	77	85	0.160262371
P38804	352.9090909	443.3333333	453.2222222	383	708.9	692.9	392.1666667	439.2	0.167210719
P38891	179.3333333	167.3125	162.9090909	218.2857143	154.9	157.8235294	210.7222222	180.375	0.750531835
P39012	854.3636364	933	1132.909091	841	2352.454545	794.5454545	1397.5	516.5	0.485053415
P39015	203.5	477	222.6666667	918	351	287.3333333	752	785	0.690787948
P39109	97	92	128	26	60.5	59	NA	NA	0.312258376
P39516	96.91666667	130.4285714	166.0833333	223.6	105.6666667	112.75	154.4285714	134.5	0.402018298
P39522	276.25	367	355.75	331.25	293.4166667	244.4166667	291.25	241.5	0.043486304
P39676	197.5	152.2857143	270.1304348	169.2857143	167.25	138.7368421	109.8333333	132.6	0.09968223
P39726	182.6	70.33333333	79.28571429	77.25	99.6	101.8571429	78.5	83.66666667	0.701939499
P39954	235.75	230.8333333	242	271.5	193.5	160.125	166.5	152.6666667	0.000940758
P39990	63.77777778	41.33333333	59.46153846	73.11111111	65.85714286	92.53846154	102.2222222	90.14285714	0.033208857
P40582	141.4444444	170.7	169.3684211	132.2	177.4210526	199.0526316	172.8	144.5555556	0.22748097
P41056	408.4444444	382.6666667	535.3333333	508.6666667	487.2222222	469.1111111	486.3333333	461.3333333	0.678225368
P41338	282.5942029	275.5185185	378.9264706	349.2	480.2463768	444.2142857	475.3703704	455.5	0.007632363
P41805	227.8823529	520.6	237.3888889	547.4	293.6111111	212.9444444	585.2	469	0.956625519
P41939	424.6071429	373.5945946	494.7261905	393.3783784	380.6547619	339.4819277	302.3333333	250.5	0.035888314
P41940	270.25	169	352.6	183.75	433.2352941	249.3	234.5	191	0.646279535
P42222	187	135	126	NA	90	123	62	44	0.046311974
P43607	180.9591837	222.25	242.6875	236.05	484.372549	462.0784314	529.6190476	450.9047619	3.24915E-05
P43616	164.8285714	156.3529412	216.1315789	183.75	222.4473684	202.9459459	154.6923077	166.1764706	0.770458799
P43620	2219.769231	1240.333333	2811.846154	1256.333333	2729.076923	2637.461538	1300.666667	1442.666667	0.797241414
P43635	382.6	477.0666667	450.6571429	447.1176471	410.8571429	390.2647059	477.5	395.8823529	0.492864002
P46367	676.5606618	687.7298578	787.0969479	768.9806763	708.1657459	674.4475138	654.2850242	645.9417476	0.124003081
P46655	224.6538462	162.1538462	273	168	191.7037037	169.96	181.9090909	125.6153846	0.2459824
P47139	435.6666667	433	544	298	607	530	492	696	0.064067285
P47176	185.6666667	130.5	73.33333333	230	72	150	239	215.5	0.787591544
P48015	270	238.25	182.4444444	270.25	140.1111111	175.625	264.5	165	0.168200852
P48164	187.5	276.8571429	245.9285714	252.5714286	261.7857143	261.5714286	298.3333333	257.8571429	0.2343825
P48415	595	399	664	292	497	378.5	232	271	0.2266

									27837
P50107	427.375	264	376.25	254	256.875	230.25	196	134	0.0533 20659
P50263	183.8	494.333 3333	235.357 1429	479.375	690.785 7143	608.5	1024.77 7778	910.666 6667	0.0110 75377
P52593	1086	407	1306	388	1149	738	551	477	0.8168 87847
P53090	144.25	213	292.75	198	123	72.3333 3333	NA	278	0.4899 31815
P53148	NA	NA	26	NA	NA	NA	NA	NA	#DIV/0 !
P53163	110.666 6667	198	141.333 3333	78	177.666 6667	119	144	177	0.4794 16441
P53184	243.25	259.4	252.5	292.8	279.857 1429	213.5	222.4	215.4	0.1843 67223
P53221	208.25	276.166 6667	280.875	404	309	302.75	337.166 6667	312.833 3333	0.6136 51615
P53228	66	114	68	94.5	56	62	176	84	0.7827 9397
P53252	148.64	159.823 5294	182.396 2264	215.875	189.666 6667	175.320 7547	212.722 2222	154.777 7778	0.7491 80278
P53312	276.202 8986	256.291 6667	321.647 8873	267.384 6154	333.075 7576	279.865 6716	290.041 6667	230.653 8462	0.9097 12208
P53598	259.114 7541	282.5	287.6	278.5	329.178 5714	291.426 2295	342.615 3846	307.884 6154	0.0276 27409
P54115	378.368 4211	457.428 5714	549.588 2353	497.142 8571	362.470 5882	290.882 3529	327.571 4286	368.142 8571	0.0257 62616
P54839	297.923 0769	243.333 3333	324.615 3846	246	274.615 3846	234.153 8462	269.666 6667	401	0.7028 83279
P81449	101.7	140.75	194.571 4286	127.8	123.666 6667	134.2	128.5	85	0.3491 32278
P81451	142.692 3077	64	244.272 7273	113.857 1429	180.833 3333	117	93.1666 6667	107.166 6667	0.7140 61683
Q00055	223.947 3684	277.3	271.205 1282	329.2	220.361 1111	207.764 7059	273.9	205.7	0.1247 50234
Q00711	306.928 5714	428.727 2727	384.413 7931	450.454 5455	285.896 5517	269	346.545 4545	349.727 2727	0.0869 98
Q00955	173.227 2727	77.8	192.772 7273	124.333 3333	141.5	122.263 1579	86.2	104.25	0.3699 68673
Q01477	116.5	119	133.5	46	125	85	35	NA	0.5348 45457
Q01560	118.5	NA	73.75	41	84.8	177	NA	NA	0.4390 63338
Q01574	650.224 4489	647.685 9903	742.751 4677	734.610 8374	745.700 3968	680.903 8076	695.801 9324	660.834 9515	0.9520 95554
Q01855	540.625	201	586.125	297	522.375	562.125	284.4	250.4	0.9915 25208
Q02207	149.6	NA	157.666 6667	101	305	287.666 6667	106	75	0.4171 14784
Q02486	280.705 8824	367	356.125	373.714 2857	447.588 2353	426.352 9412	522	482.571 4286	0.0058 9929
Q03104	181	125	262	114	199.4	253.4	81.5	139	0.9669 17246
Q03161	188.727 2727	275.125	233.333 3333	254.125	209.833 3333	192.166 6667	161.75	219.5	0.1160 13252
Q03937	149.4	91	203.666 6667	209	123.5	212.75	112	142	0.6756 8971
Q04409	222.133 3333	194.166 6667	275	190	341.5	325.416 6667	203	248	0.1820 25871

Q04438	171.375	80	186.125	68.5	370.875	335.5	129	236	0.075836292
Q04792	165.8	359	166	393.3333333	174	199.8	252	215.3333333	0.399539859
Q04947	243	108	103	162.5	81	63	145.5	74	0.155106551
Q04978	55.5	39	77	151	70	89.5	199	64	0.557358792
Q06405	433.2	586.5	500	541	432.4	442.2	374.6666667	369.5	0.033904166
Q06679	837.6666667	742	1251	1159	796.3333333	609	812	911	0.18638936
Q07478	928	281	968.5	390	745.5	723	491	385	0.792220437
Q07500	142	63.5	182	93.5	NA	50	119	79.5	0.307630062
Q07651	481.4	NA	748.6	76	1004	862.8	41	62	0.866331553
Q08245	289	92	352.5	135	272.5	238.5	54	78	0.522624627
Q08745	140.3333333	126.2857143	157.3888889	89	103.5882353	118.2941176	155	94.71428571	0.618265531
Q08969	134.8333333	NA	171.5	NA	367	313.3333333	137	157	0.214364193
Q12031	148	221	174.8	229.1666667	183	175.6	153.6666667	239.8	0.850757006
Q12165	269.4642857	289.6363636	325.4230769	262.4545455	229.28	208.9615385	220.1818182	150.3636364	0.010722836
Q12207	168.3333333	395	254.25	395	157	179.75	396	163	0.361523476
Q12213	340.8571429	244.75	372.4642857	308.75	324	295.9259259	289.125	299.4375	0.637724394
Q12230	246.5754717	242.2647059	307.2743363	281.6857143	363.4634146	359.2083333	308.575	273.275	0.081204105
Q12257	674.7	713	788.8	769	560.6	551.2	467.5	515	0.000849184
Q12289	365.08	443.2	424.08	460.5	363.92	300.04	367.9	350.9	0.026835315
Q12305	99.9	251.75	177.0909091	215.75	134.3	85.18181818	128	127.3333333	0.127340144
Q12335	344.5	394.4375	428.6388889	449.6	269.1612903	238.71875	275.5	283.0909091	0.005076762
Q12349	165	303.25	189.5714286	606.25	144.8888889	113.7	574	405	0.966091689
Q12420	2047.5	1122.2	2271.5	1229.4	2192.5	2394.5	908	980	0.923524311
Q12428	429.5625	387.3191489	507.212766	440.4042553	362.1758242	343.6276596	326.4893617	326.4888889	0.020955129
Q12672	645	874	481	820	195.6666667	199.6666667	668	454	0.067538534
Q3E792	441.4	808.3333333	565.5	797.3333333	560.1	526.5	781	570.6666667	0.700563063

[a]Average intensity calculated from all the detected peptides of proteins (calculated by R programme).

[b]T-test was calculated from average intensities of peptides of a protein from four biological replicates.

Note: T-test values less than 0.05 are marked in yellow (right most column) and protein with t-test value less than 0.05 and fold change more than 1 in all biological replicates are marked in green (left most column)

Table 7

**S. No.**	**Standard Name***	**Motif residue**	**Consensus sequence**
01	MSN2	RQRASLP	RXXXpS/pTXP
02	GLN3	RAKQTDP	RXXXpS/pTXP
03	SNF1	RSRSYP	RXXpS/pTXP
04	BCY1	REKTSTP	RXXXpS/pTXP

*Gene symbol as given in SGD (http://www.yeastgenome.org/)
